# Mass spectrometry-based analysis of rheumatoid factor

**DOI:** 10.3389/fimmu.2025.1644334

**Published:** 2025-10-10

**Authors:** Jonas De Leeuw, Birthe Michiels, Rita Derua, Tom Dehaemers, Doreen Dillaerts, Maaike Cockx, Glynis Frans, Sebastien Christian Carpentier, Patrick Verschueren, Xavier Bossuyt

**Affiliations:** ^1^ Department of Microbiology, Immunology and Transplantation, Clinical and Diagnostic Immunology, KU Leuven, Leuven, Belgium; ^2^ Department of Rheumatology, University Hospitals Leuven, Leuven, Belgium; ^3^ Department of Laboratory Medicine, University Hospitals Leuven, Leuven, Belgium; ^4^ Laboratory for Protein Phosphorylation and Proteomics, KU Leuven, Leuven, Belgium; ^5^ SyBioMa, KU Leuven, Leuven, Belgium; ^6^ PharmAbs The KU Leuven Antibody Center, Leuven, Belgium; ^7^ KU Leuven, Department of Development and Regeneration, Skeletal Biology and Engineering Research Center, Leuven, Belgium

**Keywords:** rheumatoid factor, mass spectrometry, isotypes, complementarity determining regions, immunoglobulins

## Abstract

**Introduction:**

Rheumatoid factor (RF) are autoantibodies that are found in approximately two thirds of patients with rheumatoid arthritis, a chronic autoimmune disease characterized by potentially destructive inflammation of the joints. RF consists of polyclonal antibodies targeting the Fc part of immunoglobulin G. Despite its clinical relevance, RF is not specific for RA, and conventional assays for RF detection, predominantly solid-phase tests detecting IgM RF, suffer from poor harmonization and the disability to test more than one RF isotype.

**Methods:**

We studied RF using a mass-spectrometry-based approach in RF(+), RF(–) rheumatoid arthritis patients and in disease controls. This allowed evaluation of RF at the amino acid level, including the variable and hypervariable region part of RF. RF was captured on Fc coated microwell plates, isolated, digested into peptides and analyzed by liquid chromatography tandem mass spectrometry. An initial proof-of-concept analysis was conducted comprising 12 samples, followed by a larger-scale experiment comprising 86 samples.

**Results:**

Principal component analysis and sparse partial least squares discriminant analysis demonstrated that RF(+) RA patients displayed peptides that were differentially expressed compared with disease control patients. Framework region-derived peptides, variable region-derived peptides as well as de novo sequenced peptides not present in the human proteome database, were found to be enriched in RF(+) sera compared to disease control sera. Interestingly, some of these peptides were also upregulated in sera from RF(–) RA patients. Furthermore, mass spectrometry analysis revealed different RF isotypes. In addition to IgM, also IgA and IgG isotypes were observed. RF-IgG2 isotype was observed in RF(+) as well as in RF(–) RA patients.

**Discussion:**

In summary, our findings highlight that mass spectrometry provides a platform for elucidating the heterogeneity and isotypic diversity of RF autoantibodies in RA, overcoming limitations inherent to current solid-phase RF assays. Upregulated de novo peptides were found, possibly related to the hypervariable regions of RF. Further validation using integrated proteomic and genomic approaches is required to confirm these novel peptides and their localization within the RF hypervariable regions.

## Introduction

Rheumatoid arthritis (RA) is a chronic autoimmune disease characterized by joint inflammation. It is a relatively common disease, affecting up to 1% of the population in Europe (1). RA is driven by multiple pathophysiological factors and manifests with high heterogeneity, both between and within patients along the disease course. If left untreated, severe joint inflammation or destruction leads to impaired physical function and potentially workplace disability (2). Rheumatoid factor (RF) and anti-cyclic citrullinated peptide (anti-CCP) antibodies are biomarkers for RA and are part of the classification criteria issued by the American College of Rheumatology (ACR) and the European Alliance of Associations for Rheumatology in 2010 (3). However, the applicability of RF and anti-CCP for RA diagnosis and prognosis is limited by the fact that these markers are absent in approximately one-third of patients with RA (seronegative RA), a percentage that is even higher in early RA (4). The presence of RF not only helps the diagnostic process but also predicts a more severe disease course, with more radiographic damage and systemic involvement in RF-positive compared to RF-negative RA patients (5).

RFs are polyclonal autoantibodies directed against the constant Fc part of immunoglobulin G (IgG). In clinical laboratories, RF is measured using nephelometry or latex agglutination, which detects all RF isotypes without the ability to differentiate between isotypes. Alternatively, solid-phase assays, such as enzyme-linked immunosorbent assay (ELISA) and fluorescence enzyme immunoassay (FEIA), enable the detection of isotype-specific RF (6). IgM is the most prevalent RF isotype, followed by IgG and IgA. A recent systematic review and meta-analysis showed that testing all RF isotypes using nephelometry or latex agglutination had the highest sensitivity for RA, that determination of the IgA isotype had the highest specificity, and that determination of the IgM isotype had the highest diagnostic odds ratio (6). A recent multicenter study confirmed that the sensitivity of IgA-RF for diagnosing RA was lower than the sensitivity of IgM-RF and found that double positivity for IgM-RF and IgA-RF had a higher specificity for RA than either IgM-RF or IgA-RF (7). Similar to high-titer IgM-RF, IgA-RF correlates with the severity of RA, namely, disease progression, erosions, and extra-articular manifestations (8–10) with poor response to TNF-blocking biologicals (11). Furthermore, current standardized RF testing using different clinically available assays is suboptimal, resulting in differences between assays in classifying patients using the classification criteria (12, 13). To conclude, there is room for improvement in clinical RF testing.

Little is known about the antigen-binding (Fab) regions of polyclonal autoantibodies in general, including RF. The amino acid sequence variation in the variable domains of an immunoglobulin (Ig) is mainly confined to three small hypervariable loops that determine antigen specificity by forming a surface complementary to the target epitope of the antigen, more commonly termed complementarity-determining regions (CDRs). The enormous antibody diversity originates from B-cell development during which a large combinatorial diversity in antibodies is created through VDJ recombination, junctional diversity, and somatic hypermutation (14–16). In paraneoplastic disease, chronic inflammatory demyelinating polyradiculoneuropathy, and lung cancer, it has been suggested that specific peptides in the Fab region are shared between individuals who have antibodies targeting the same antigen (17–19).

The aim of the present study was to better understand the nature of RF at the protein level in a population of patients diagnosed with RA. This was achieved through the application of a mass spectrometry-based approach following the isolation of RF, facilitating a comprehensive analysis of RF in its entirety. This approach not only enables the differentiation of RF isotypes, which is not feasible using a conventional single solid-phase test, but also permits sequencing of RF. This provides an opportunity to characterize the variable region sequences of RF, which remain undocumented in human proteome databases.

## Materials and methods

### Ethical approval and patient samples

The study was approved by the ethics committee of the University Hospitals Leuven (S65091). The individuals included in this study were disease-modifying antirheumatic drug (DMARD)-naïve early RA patients from the CareRA cohort (S51411). The diagnosis of RA was based on the 1987 ACR classification criteria (20). Disease controls were patients who consulted a rheumatologist at the University Hospitals Leuven and, after rheumatological workup, had no autoinflammatory or autoimmune disease. RF was measured using nephelometry. Patient characteristics are listed in **Supplementary Tables 1** and **2**.

### Reagents

Human Fc IgG1 was purchased from Invitrogen (Thermo Fisher Scientific, Massachusetts, USA, ref A42561), and Tris-HCl was purchased from Invitrogen. Dithiothreitol (DTT), iodoacetamide (IAM), and urea were purchased from Sigma-Aldrich, Massachusetts, USA, digestion enzymes endoproteinase Lys-C and chymotrypsin from Thermo Fisher Scientific, and mass spectrometry (MS)-grade formic acid and acetonitrile from Biosolve, Valkenswaard, Netherlands.

### RF enrichment

Costar plates were coated with Fc IgG1 (0.5 µg per well) and left overnight at 4°C. The following day, coated plates were washed three times with PBS-T [0.05% Tween-20 in phosphate buffered saline (PBS)]. Diluted serum (1/10 in PBS-T) was added, and the plates were incubated in a thermomixer [Retention time (RT), 800 rpm, 1 h]. After incubation, plates were washed two times with PBS-T and five times with PBS. Finally, plates were washed with 50 mM Tris-HCl.

### Digestion with chymotrypsin/lysin C

Following enrichment, 60 µL of 4 M urea/50 mM Tris-HCl was added to each well. Afterward, proteins were reduced for 1 h in a thermomixer (800 rpm, 37°C) in the presence of 8 mM DTT, followed by alkylation for 30 min in the dark (37°C) in the presence of 22 mM IAM. Digestion was started by the addition of Lys-C at 0.4 µg/well and left overnight (thermomixer 37°C, 800 rpm, dark). The next day, 50 mM Tris-HCl was added until the urea concentration dropped below 1 M. Chymotrypsin was added at 1 µg/well, and digestion of the proteins continued in the thermomixer (4 h, 37°C, 800 rpm). Finally, digestion was stopped in the presence of 1% formic acid (FA).

### Desalting

Desalting was performed on Sep-Pak 96-well tC18 µElution plates using a vacuum pump. To activate the column, 200 µL 50% acetonitrile (ACN) was added to each well, and the pump was activated for 1 min. This activation step was repeated once. For equilibration, 200 µL of 0.5% FA/5% ACN was added to each well, and the pump was activated for 1 min. This equilibration step was repeated once. Next, samples were loaded on the column, and the pump was activated. The flow-through was passed through the column again to ensure maximum binding. Afterward, the column was washed four times with 0.5% FA/5% ACN. To elute the samples, 50 µL of 70% ACN was loaded on the column, the pump was activated, and the flow-through was collected in a new 96-well plate. The samples were transferred to 1.5-mL Eppendorf tubes and dried in the SpeedVac centrifuge (Uniequip Univapo 150 ECH). Dried samples were stored at −20°C.

### MS analysis

The dried samples were resuspended in 15 µL of 0.1% FA/5% ACN before being injected (5 μL) and separated on an Ultimate 3000 UPLC system (Dionex, Thermo Fisher Scientific) equipped with an Acclaim PepMap 100 pre-column (C18 3 μm–100 Å, Thermo Fisher Scientific) and a C18 PepMap RSLC (2 μm, 50 μm–15 cm, Thermo Fisher Scientific) using a linear gradient (300 nL/min) of 0%–4% buffer B (80% ACN, 0.08% FA) in 3 min, 4%–10% B in 7 min, 10%–35% in 25 min, 35%–38% in 5 min, 38%–40% in 2 min, 40%–65% in 5 min, 65%–95% in 1 min, 95% for 9 min, 95%–5% in 1 min, and 5% for 9 min.

The Q Exactive Orbitrap mass spectrometer (Thermo Fisher Scientific) was operated in positive ion mode using data-dependent acquisition with a survey MS scan at a resolution of 70,000 [Full Width at Half Maximum (FWHM) at m/z 200], followed by tandem mass spectrometry (MS/MS) scans (resolution 17,500) of the top 10 most intense peaks with +2, +3, +4, and +5 charged ions above a threshold ion count of 16,000 using normalized collision energy (NCE) of 25 eV with an isolation window of 2.0 m/z, apex trigger of 5–15 s, and dynamic exclusion of 30 s. All data were acquired using the Xcalibur 3.1.66.10 software (Thermo Fisher Scientific).

### Bioinformatics analysis

The Progenesis software (version 4.1; Nonlinear Dynamics Ltd., Newcastle, UK) was used for relative quantification of data. To correct for possible sample variation, the samples were normalized based on the detected abundance values (median and median absolute deviation outlier filtering approach, https://www.nonlinear.com/progenesis/, Nonlinear Dynamics Ltd., Newcastle, UK). Mascot (version 2.2.06; Matrix Science Inc., London, UK) was used for the identification of peptides by searching against the UniProt (Swiss-Prot + TrEMBL) Homo sapiens database (194,319 entries, version 02/03/2021).

Additionally, *de novo* sequencing and parallel database searching using the PEAKS Studio software (version 12.5; Bioinformatics Solutions Inc., Waterloo, ON, Canada) were performed. Database searching was performed using the UniProt (Swiss-Prot) Homo sapiens database (20,434 entries, version 20/03/2024) with a peptide false discovery rate (FDR) of 1%. Since protein quantification using the PEAKS software only takes into account unique peptides per protein, the reviewed Swiss-Prot database was used (instead of Swiss-Prot + TrEMBL), providing more accurate protein quantification due to less protein redundancy. All Ig-related peptides identified using Mascot with a Mascot score ≥30 were excluded for further *de novo* sequencing. A *de novo* score [average of local confidence (ALC%)] was assigned by PEAKS based on the reliability of each amino acid in the *de novo* sequence. Only *de novo* peptides with ALC% scores >80% and with a sequence consisting of three or more amino acids were included for further analysis. Conflicting peptide identifications arising from parallel database searches using Mascot and PEAKS were resolved by prioritizing the Mascot identification when the Mascot score was ≥30. In cases of a Mascot score <30, the PEAKS identification was retained. All database hits related to the constant region of different Ig isotypes were extracted. Since peptides present in more than one protein were excluded from the PEAKS protein quantification, unique peptides for each isotype were taken into account for quantification. The Z-score (standardized normalized abundance) of the Ig-related protein accessions was calculated to illustrate protein abundance in heatmaps constructed using the R (version 4.20.0) package ComplexHeatmap (version 2.12.1). An overview of the used settings in the database searches is given in **Supplementary Table 3**.

*De novo* peptide sequences and peptide sequences with a Mascot score <30 were subsequently aligned to databases containing V-, D-, or J-region germline sequences derived from the IMGT database (21) using the IgBLAST (22) and IMGT/DomainGapAlign (23) algorithms. Peptides with sufficient match to the human Ig V-region databases were assigned to a frame region (FR) or CDR of the corresponding Ig germline gene (IgBLAST criteria for FR and CDR1/2: E-value ≤1; IMGT criteria: E-value ≤1, Smith–Waterman score >30, and min. 3 amino acids aligned to any CDR). An overview of the bioinformatics analysis workflow is given in **Supplementary Figure 1**.

### Statistical analysis

Normalized abundance of peptides was used to perform the Kruskal–Wallis testing, followed by a *post-hoc* Dunn test to acquire p-values on the peptide and protein levels. Multiple correction testing adjustment was performed using the Holm–Šídák method on the peptide level, and adjusted p-values were reported where applicable. The mean abundance of peptides in RA patients and control patients was calculated and used to calculate fold change (FC). Volcano plots for peptides were constructed using log2(fold change) and −log10(adjusted p-value) using the R (version 4.20.0) package ggplot2 (version 3.5.0).

Principal component analysis (PCA) and sparse partial least squares discriminant analysis (sPLS-DA) of the normalized abundance values of the identified Ig and variable region peptides were performed to identify the most predictive/discriminative peptides to classify different patient groups (24, 25). Three steps were performed for each experiment in the sPLS-DA. First, sPLS-DA models require X and Y matrices as input. For each experiment, the normalized abundance values per peptide for each individual were used as the X matrix, and all different patient groups were used as a categorical group label in the Y matrix. Second, two sPLS-DA parameters were tuned based on the balanced error rate and maximum distance metric: the optimal number of components (H) and the optimal number of variables (=peptides) to select each component. Third, sPLS-DA models were calculated for H components (min 2 and max 5) with the component-specific optimal numbers of peptides using threefold cross-validation with 50 repeats. Peptides with the lowest median Z-score in the disease control group, with a statistical significance defined as having an adjusted p-value of <0.05, an FC > 5, and an occurrence of >50% after 50 repeats and evaluated to be related to the variable region of RF, were labeled as “discriminative peptides”. sPLS-DA was performed using the MixOmics package (version 6.20.0) in R. Peptides detected in two experiments were plotted using their median grouped abundance in a heatmap constructed using GraphPad Prism 9 (version 9.2.0).

## Results

### Proof-of-concept experiment

**Figure 1:**
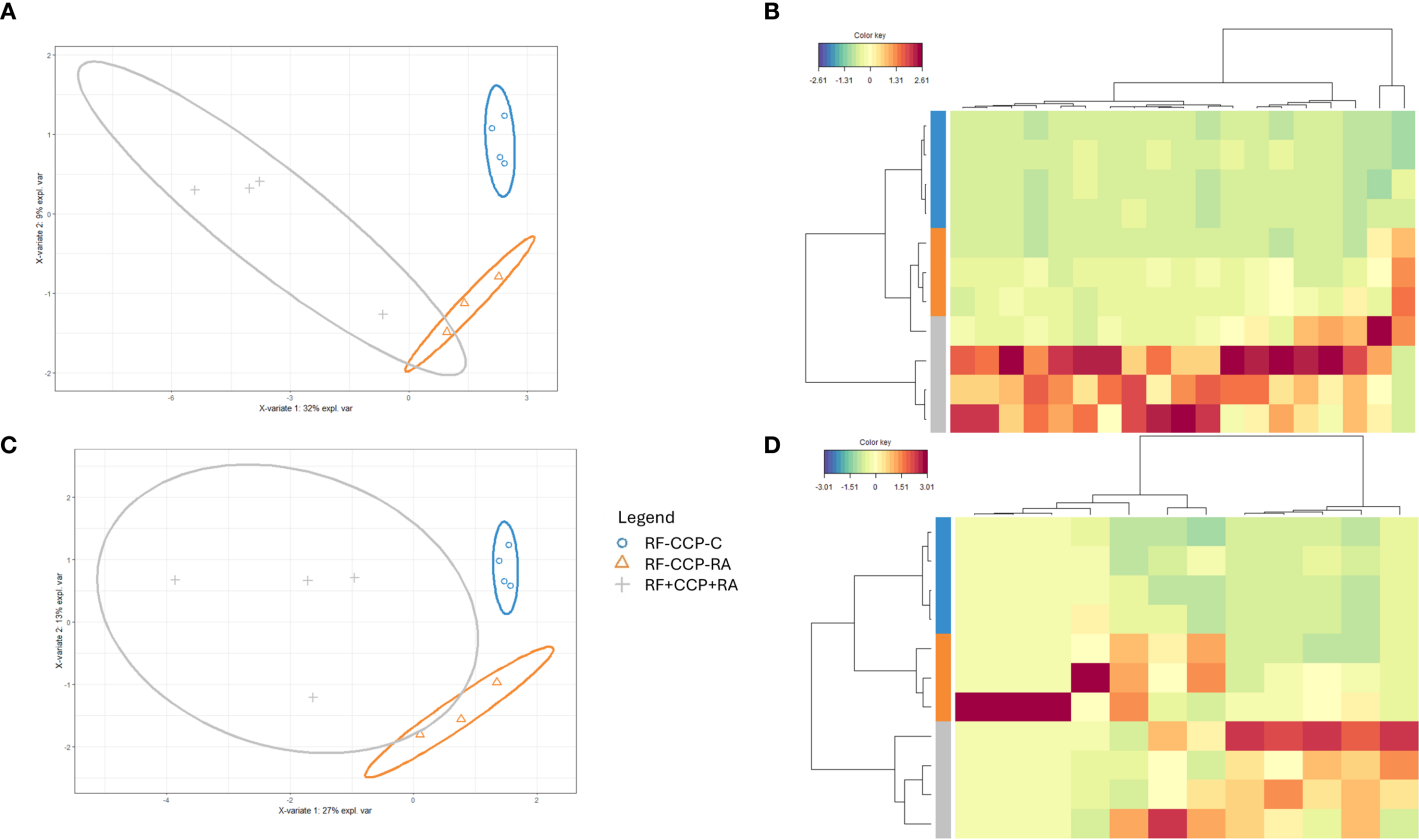
Sparse partial least squares discriminant analysis (sPLS-DA) reveals distinct rheumatoid factor (RF) Ig peptide repertoires between rheumatoid arthritis (RA) and controls (proof-of-concept experiment). RF from four RF(+)/anti-cyclic citrullinated peptide (anti-CCP)(+) RA patients, three RF(−)/anti-CCP(−) RA patients, and four RF(−)/anti-CCP(−) disease controls was isolated, digested into peptides, and analyzed using liquid chromatography tandem mass spectrometry (LC-MS/MS). Colors were assigned for different groups and consistently applied across the heatmaps and sPLS-DA figures. **(A)** sPLS-DA displaying the variance of the most discriminant peptides when comparing all Ig features in RF(+)/anti-CCP(+) RA patients, RF(−)/anti-CCP(−) RA patients, and RF(−)/anti-CCP(−) disease control patient samples. **(B)** Heatmap after hierarchical clustering of the features identified as being most discriminant within the Ig repertoire using sPLS-DA. Heatmap color key indicates the difference in Z-score (standardized normalized abundance). **(C)** sPLS-DA plotting the variable region repertoire based on the most discriminating features in RF(+)/anti-CCP(+) RA patients, RF(−)/anti-CCP(−) RA patients, and RF(−)/anti-CCP(−) disease control patient samples. **(D)** Heatmap after hierarchical clustering of the features identified as being most discriminant within the variable region repertoire using sPLS-DA. Heatmap color key indicates the difference in Z-score.

In order to study RF at the protein sequence level, RF was isolated (by binding to an Fc IgG1-coated ELISA plate), digested into peptides, and subsequently analyzed using liquid chromatography MS/MS (LC-MS/MS). A first experiment was performed with 12 patient samples: four RF(+)/anti-CCP(+) RA patients, four RF(−)/anti-CCP(−) RA patients, and four disease control patients. A summary of the data statistics of the MS results is given in **Supplementary Figures 2** and **3**. One RF(−)/anti-CCP(−) RA patient was excluded due to technical difficulties with the MS analysis. Ig-related peptide sequences, including Ig variable region peptide sequences, were identified through database search and *de novo* sequencing according to the workflow described in the Materials and Methods. PCA plots analyzing all Ig-related peptides and the variable region of Ig-related peptides are shown in **Supplementary Figure 4**; sPLS-DA plots for Ig-related peptides and the variable region of Ig-related peptides are shown in **Figure 1**. PCA showed that RF-positive samples clustered separately from the RF-negative samples, although with a high heterogeneity in peptide expression within the RF(+) group. sPLS-DA showed that the three different patient groups clustered separately. Model performance plots regarding PCA and sPLS-DA are shown in **Supplementary Figures 5** and **6**.

#### RF isotypes in the proof-of-concept experiment

**Figure 2:**
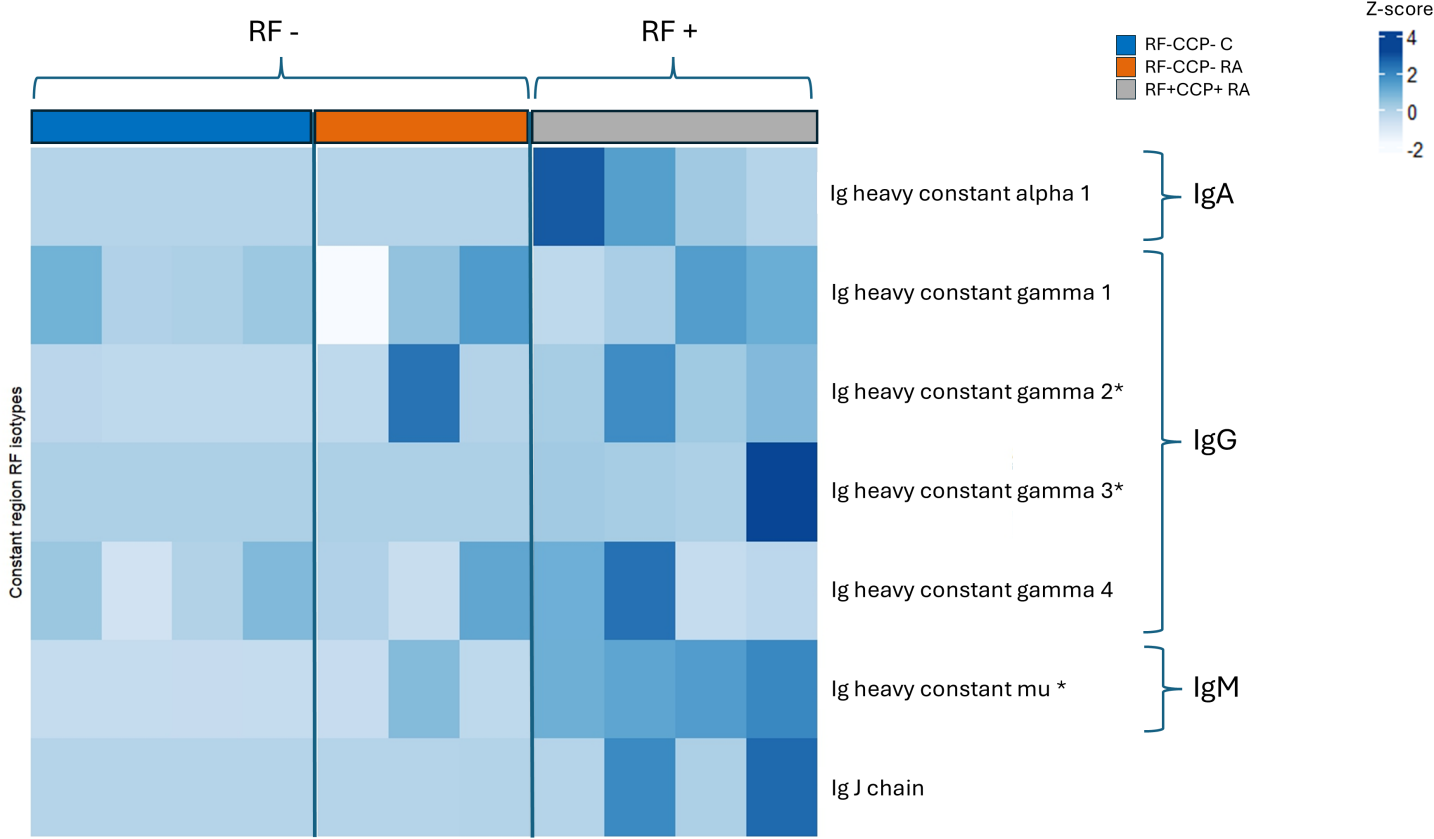
Heatmap representing Z-scores of different rheumatoid factor (RF) isotypes in rheumatoid arthritis (RA) patients and controls (proof-of-concept experiment). Comparison of RF isotypes in four RF(+)/anti-cyclic citrullinated peptide (anti-CCP)(+) RA patients, three RF(−)/anti-CCP(−) RA patients, and four RF(−)/anti-CCP(−) disease controls after isolation, digestion into peptides, and analysis by liquid chromatography tandem mass spectrometry (LC-MS/MS). Data shown are the Ig isotypes (IgA, IgG subclasses, and IgM), quantified at the protein level based on unique peptides per protein of the Fc Ig part. *p <0.05 using Kruskal–Wallis testing followed by *post-hoc* Dunn’s test when comparing RF(+)/anti-CCP(+) RA patient samples with RF(−)/anti-CCP(−) disease control samples.

The isotype distribution of the isolated RFs is illustrated in **Figure 2**. Remarkably, in sera of seronegative RA patients, IgG1-RF, IgG2-RF, IgG3-RF, and IgM-RF were identified with a low abundance. IgA-RF antibodies were identified in an RF-positive sample, but not in patients with seronegative RA or in controls.

### Main experiment

**Figure 3:**
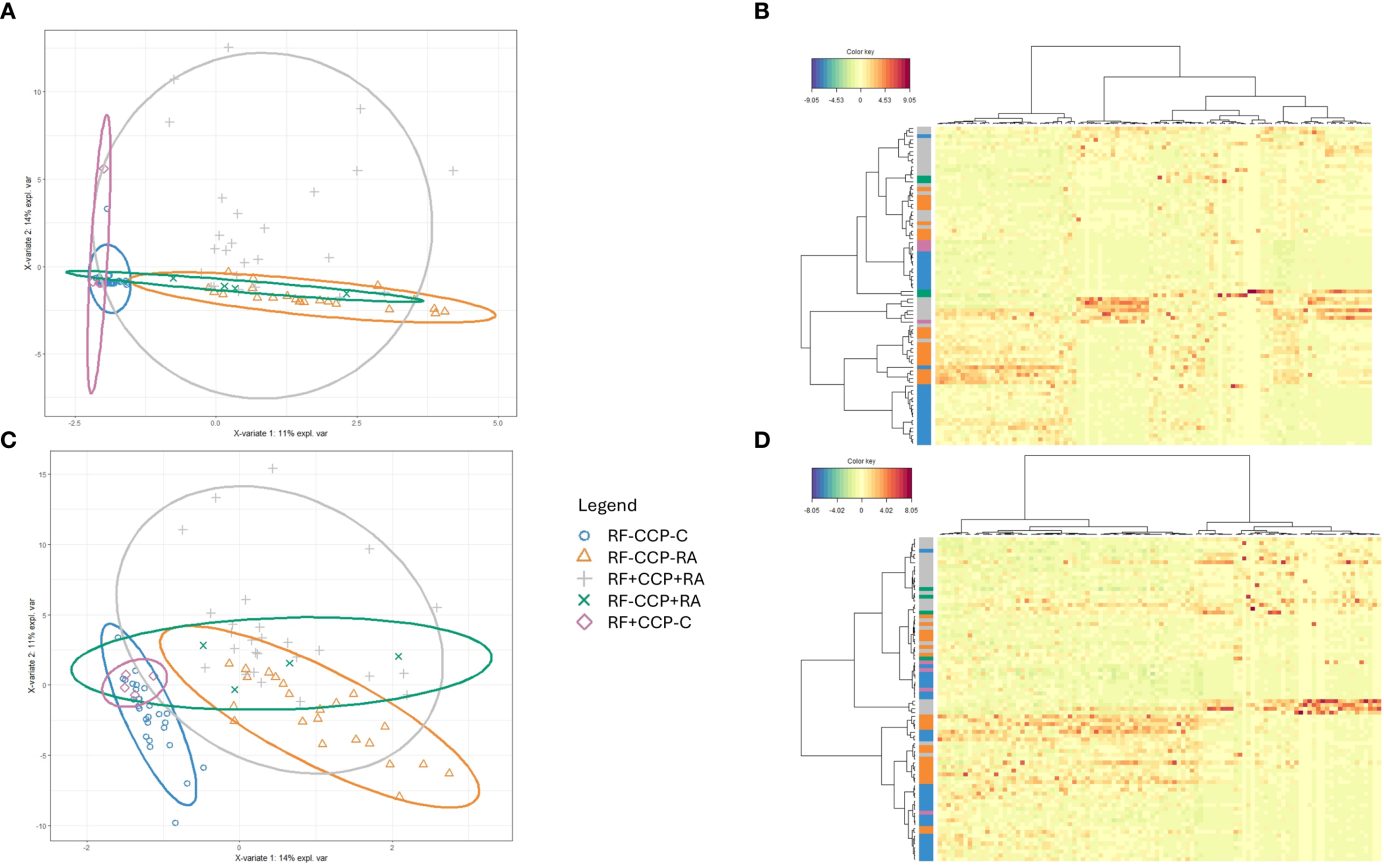
Sparse partial least squares discriminant analysis (sPLS-DA) of rheumatoid factor (RF)-derived peptides in rheumatoid arthritis (RA) and controls. RF from 27 RF(+)/anti-cyclic citrullinated peptide (anti-CCP)(+) RA patients, five RF(−)/anti-CCP(+) RA patients, 22 RF(−)/anti-CCP(−) RA patients, 28 RF(−) disease controls, and four RF(+) disease controls was isolated, digested into peptides, and analyzed using liquid chromatography tandem mass spectrometry (LC-MS/MS). Colors were assigned for different groups and consistently applied across the heatmaps and sPLS-DA figures. **(A)** sPLS-DA plotting the Ig repertoire based on the most discriminating peptides in sera from different groups. **(B)** Heatmap after hierarchical clustering of the features identified as being most discriminant within the Ig repertoire using sPLS-DA. Heatmap color key indicates the difference in Z-score. **(C)** sPLS-DA plotting the variable region repertoire based on the most discriminating peptides in sera from different groups. **(D)** Heatmap after hierarchical clustering of the features identified as being most discriminant within the variable region repertoire using sPLS-DA. Heatmap color key indicates the difference in Z-score.

In a second larger experiment, 86 samples (all patients were different from those in the first experiment) were included, comprising 28 RF(−) disease controls, four RF(+) disease controls, 22 RF(−)/anti-CCP(−) RA patients, 27 RF(+)/anti-CCP(+) RA patients, and five RF(−)/anti-CCP(+) RA patients. A summary of the data statistics of the MS results is given in **Supplementary Figures 7** and **8**. **Figure 3** and **Supplementary Figure 9** illustrate sPLS-DA and PCA for all Ig-related peptides and only Ig-related variable region peptides, respectively. Similar to the initial experiment, PCA indicated that RF(+) RA patients tended to separate from the controls. sPLS-DA indicated that RF(+) as well as RF(−) RA patients clustered away from the controls, with more variability in the RF(+) RA group. Model performance plots are provided in **Supplementary Figures 10** and **11**.

**Figure 4:**
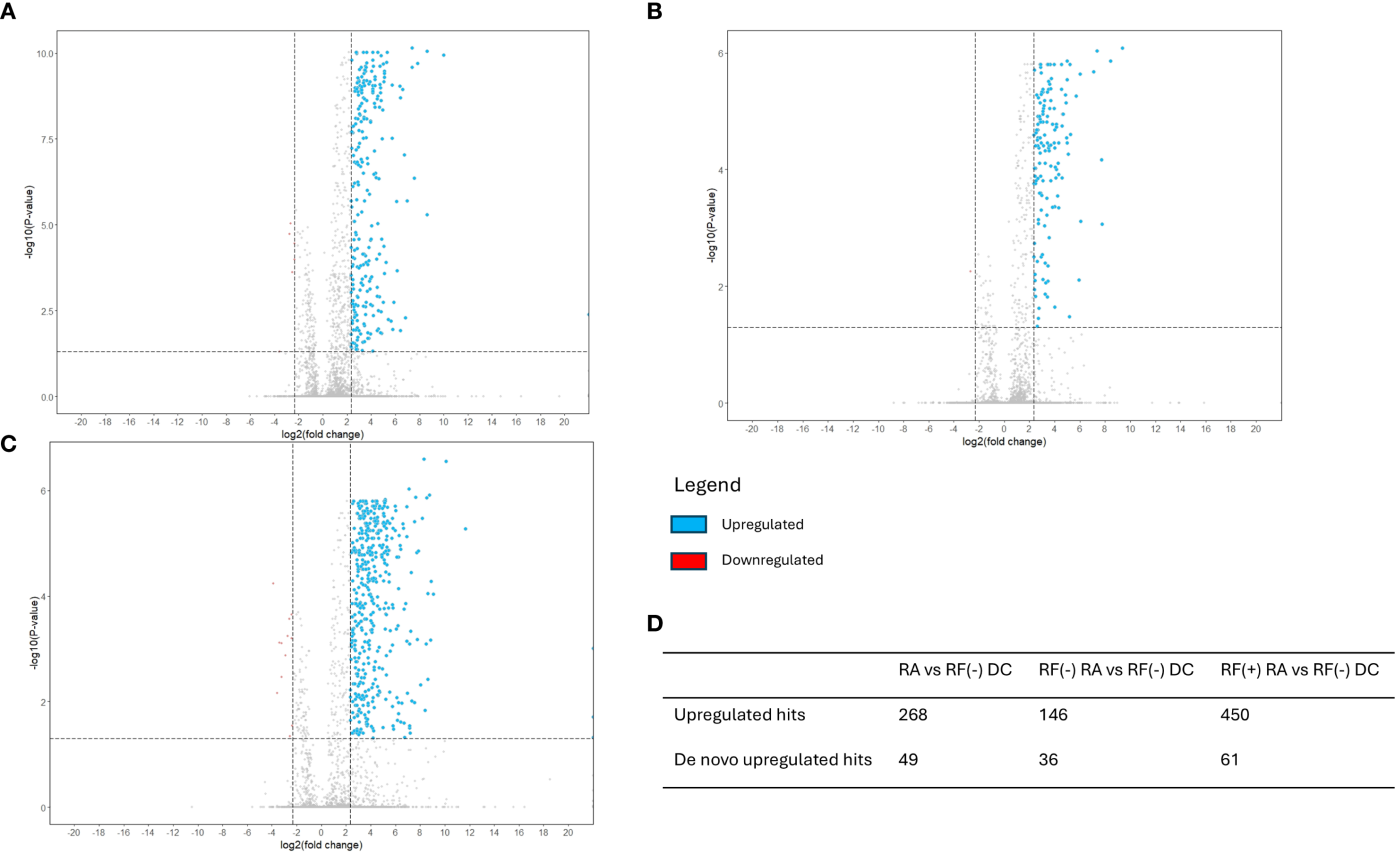
Volcano plots reveal upregulation of a set of rheumatoid factor (RF)-derived peptides in RF(+) and RF(−) rheumatoid arthritis (RA) versus RF(−) disease controls. RF from 27 RF(+)/anti-cyclic citrullinated peptide (anti-CCP)(+) RA patients, five RF(−)/anti-CCP(+) RA patients, 22 RF(−)/anti-CCP(−) RA patients, 28 RF(−)/anti-CCP(−) disease controls, and four RF(+)/anti-CCP(−) disease controls was isolated, digested into peptides, and analyzed using liquid chromatography tandem mass spectrometry (LC-MS/MS). Sequenced features (Ig-related and non-Ig-related features) were incorporated in the volcano plots with log2(fold change) on the x-axis and −log10(adjusted p-value) on y-axis. Upregulated peptides in the RA group, defined as having an adjusted p-value <0.05 and an FC > 5, are presented in blue. Downregulated peptides, defined as an adjusted p-value <0.05 with FC < 0.2, are presented in red. Vertical dotted line represents FC of 0.2 and 5; the horizontal dotted line represents an adjusted p-value of 0.05. **(A)** Volcano plot comparing seropositive and seronegative RA patient samples versus disease controls. **(B)** Volcano plot comparing RF(−) RA patient samples versus disease controls. **(C)** Volcano plot comparing RF(+) RA patient samples versus disease controls. **(D)** Table summarizing upregulated hits when comparing different patient groups.

**Figure 5:**
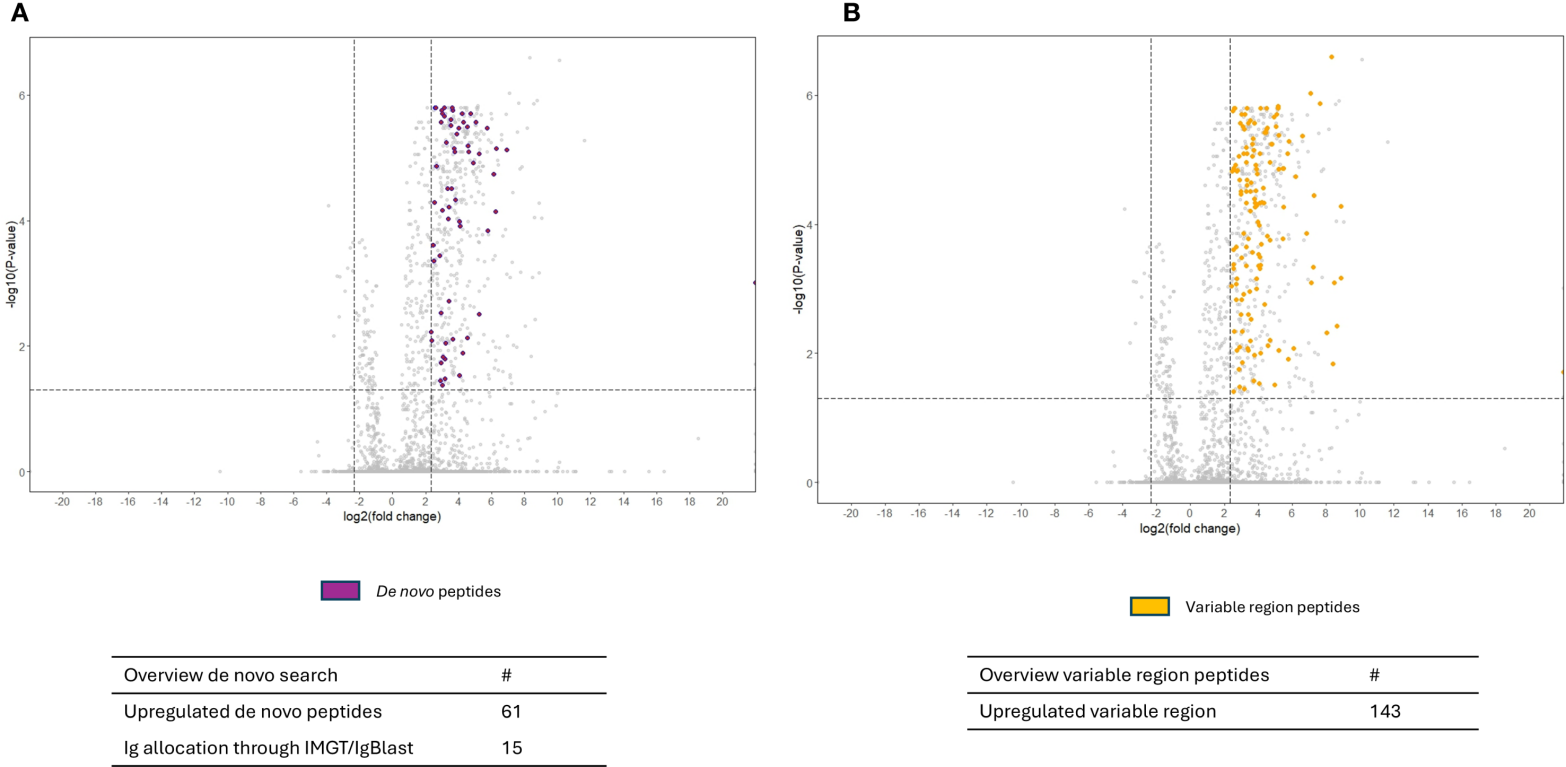
Volcano plots comparing rheumatoid factor (RF)-derived peptides from RF(+)/anti-cyclic citrullinated peptide (anti-CCP)(+) patient samples with RF(−)/anti-CCP(−) control samples, with annotation of *de novo* hits and variable region hits in the upregulated peptides. RF from 27 RF(+)/anti-CCP(+) rheumatoid arthritis (RA) patients, five RF(−)/anti-CCP(+) RA patients, 22 RF(−)/anti-CCP(−) RA patients, 28 RF(−)/anti-CCP(−) disease controls, and four RF(+)/anti-CCP(−) disease controls was isolated, digested into peptides, and analyzed using liquid chromatography tandem mass spectrometry (LC-MS/MS). Sequenced features (Ig-related and non-Ig-related features) were incorporated in the volcano plots with log2(fold change) on the x-axis and −log10(adjusted p-value) on y-axis. Shown are volcano plots comparing RF(+)/anti-CCP(+) patient samples with RF(−)/anti-CCP(−) control samples. Vertical dotted lines represent FC of 0.2 and 5, and horizontal dotted line represents an adjusted p-value of 0.05. Features were considered upregulated when having an FC > 5 and an adjusted p-value <0.05. **(A)** The upregulated *de novo* peptides that could be identified through *de novo* sequencing are annotated in purple. **(B)** Upregulated variable region peptides identified through a Mascot database search, annotated in yellow.

**Figure 6:**
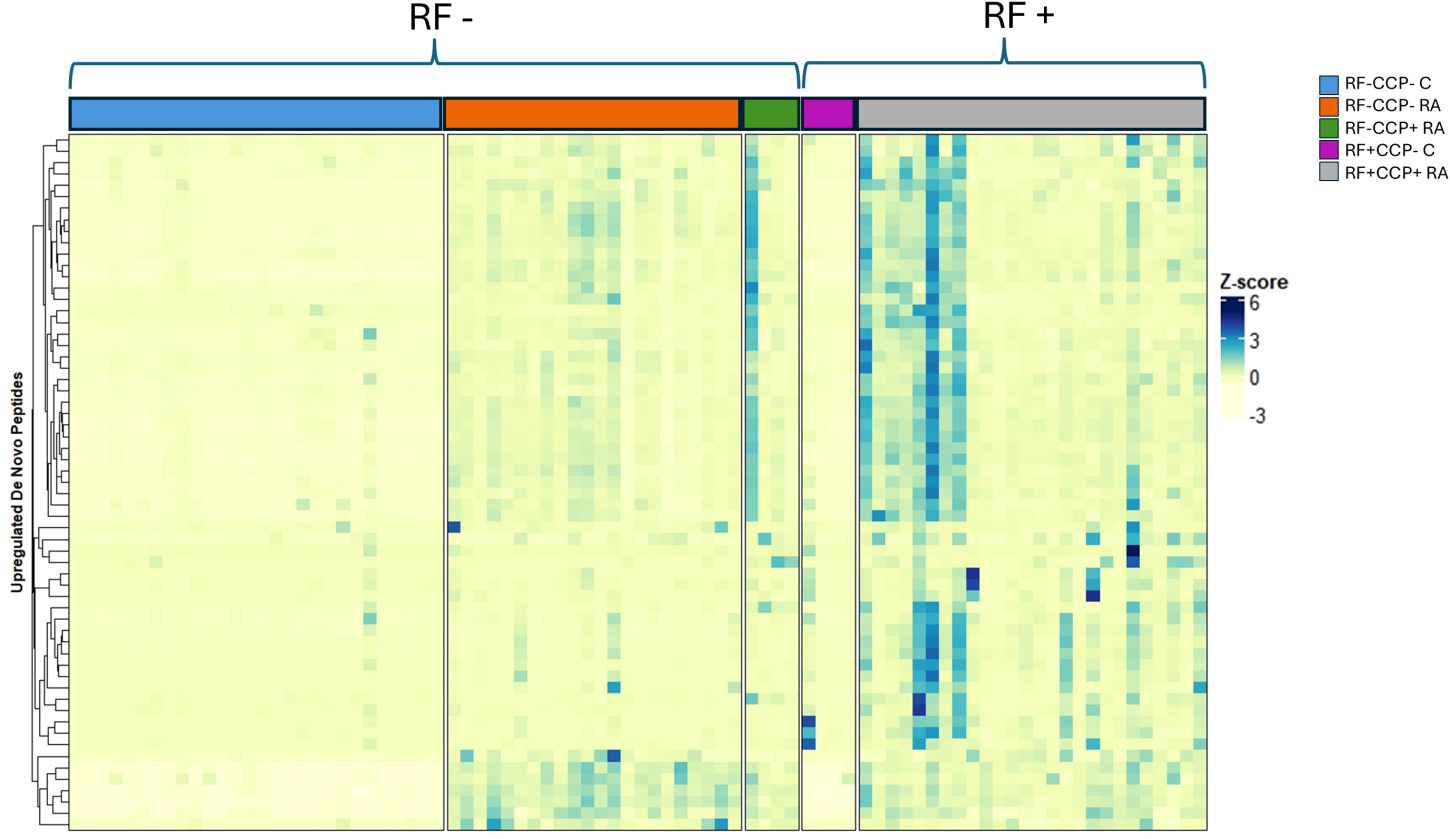
Heatmap illustrating *de novo* upregulated rheumatoid factor (RF)-derived peptides overexpressed in RF(+)/anti-cyclic citrullinated peptide (anti-CCP)(+) rheumatoid arthritis (RA) patients compared to RF(−)/anti-CCP(−) disease controls, with hierarchical clustering of peptides based on their Z-scores. RF from 27 RF(+)/anti-CCP(+) RA patients, five RF(−)/anti-CCP(+) RA patients, 22 RF(−)/anti-CCP(−) RA patients, 28 RF(−) disease controls, and four RF(+) disease controls was isolated, digested into peptides, and analyzed using liquid chromatography tandem mass spectrometry (LC-MS/MS). The heatmap illustrates the Z-scores of the upregulated *de novo* peptides. Peptides were considered upregulated when having an adjusted p-value <0.05 and an FC > 5 when comparing RF(+)/anti-CCP(+) RA patients with RF(−)/anti-CCP(−) disease controls. A full list of *de novo* sequences with their associated mass-to-charge values, charges, retention time, FC, and adjusted p-values is given in **Supplementary Table 4**.

**Figure 7:**
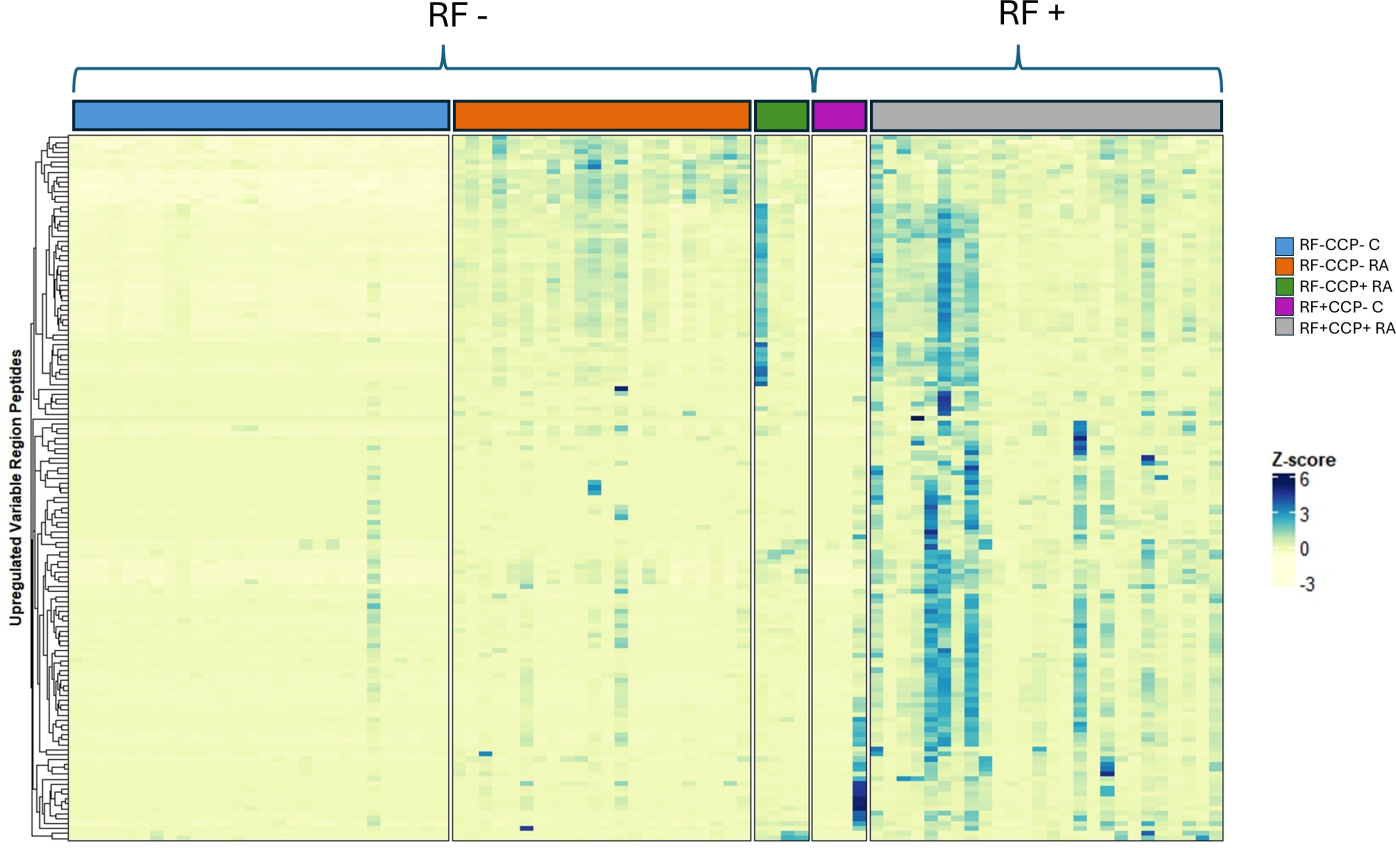
Heatmap illustrating rheumatoid factor (RF)-derived variable region peptides overexpressed in RF(+)/anti-cyclic citrullinated peptide (anti-CCP)(+) rheumatoid arthritis (RA) patients compared to RF(−)/anti-CCP(−) disease controls, with hierarchical clustering of peptides based on their Z-scores. RF from 27 RF(+)/anti-CCP(+) RA patients, five RF(−)/anti-CCP(+) RA patients, 22 RF(−)/anti-CCP(−) RA patients, 28 RF(−) disease controls, and four RF(+) disease controls was isolated, digested into peptides, and analyzed using liquid chromatography tandem mass spectrometry (LC-MS/MS). The heatmap illustrates the Z-score of the upregulated variable region peptides. Peptides were considered upregulated when having an adjusted p-value <0.05 and an FC > 5 when comparing RF(+)/anti-CCP(+) RA patients with RF(−)/anti-CCP(−) disease controls. All peptides were identified through a Mascot database search. A full list of variable region sequences with their associated mass-to-charge values, charges, retention time, FC, and adjusted p-values is given in **Supplementary Table 5**.

Volcano plots illustrating sequenced peptides, through either database search or *de novo* sequencing, are shown in **Figure 4**. When comparing RF(+)/anti-CCP(+) RA patients with RF(−) disease controls, 450 peptides with an adjusted p-value <0.05 and an FC > 5 were found. When comparing RF(−)/anti-CCP(−) RA patients with RF(−) disease controls, 146 peptides with an adjusted p-value <0.05 and an FC > 5 were found. Of the 450 peptides upregulated in RF(+)/anti-CCP(+) RA patients, 61 were sequenced through *de novo* sequencing, of which 15 were allocated to variable regions through IgBlast or IMGT. Volcano plots depicting the *de novo* and variable region peptides are illustrated in **Figure 5**. A heatmap illustrating the Z-scores per patient group for the upregulated *de novo* sequences is given in **Figure 6**. A full list of the *de novo* sequenced peptides upregulated in RF(+)/anti-CCP(+) RA patients and RF(+)/anti-CCP(+) RA patients can be found in **Supplementary Table 4**. Note that some of the *de novo* peptides have similar sequences with marginally different retention times. Out of the 450 identified upregulated peptides in RF(+)/anti-CCP(+) RA patients, 143 were allocated to the variable part of immunoglobulins through a Mascot database search. Most of these were allocated to framework regions or to (part of the) framework region and CDR. **Figure 7** shows a heatmap of the variable region peptides with Z-scores per patient group. A full list of the upregulated variable region peptides (with their relevant characteristics) in RF(+)/anti-CCP(+) RA patients and RF(−)/anti-CCP(+) RA patients can be found in **Supplementary Table 5**.

Out of the 143 upregulated variable peptides in the main experiment, 22 were found to be upregulated (adjusted p-value <0.05 and FC > 5) in RF(+)/anti-CCP(+) RA patients compared to RF(−)/anti-CCP(−) disease controls in the proof-of-concept experiment. **Supplementary Table 6** lists these peptides with their relevant characteristics.

**Figure 8:**
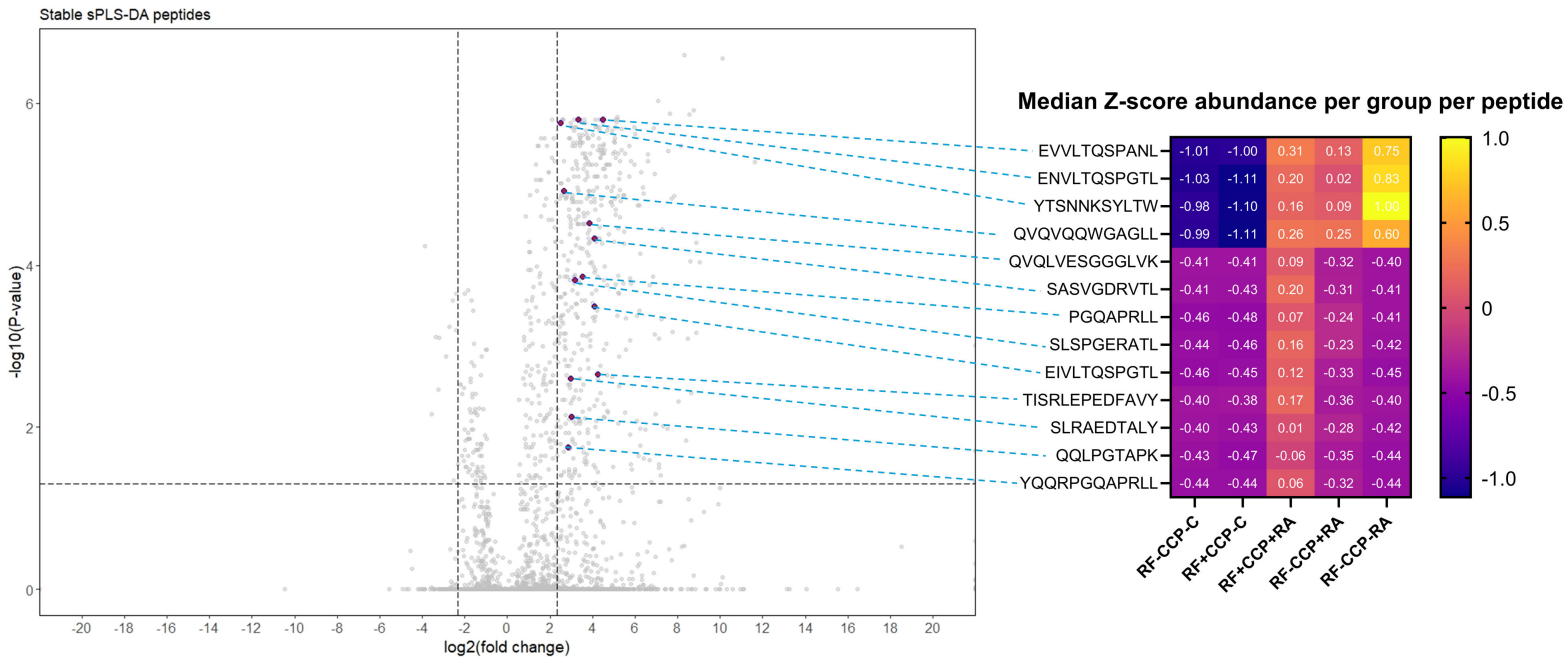
Volcano plot comparing rheumatoid factor (RF)-derived peptides from RF(+)/anti-cyclic citrullinated peptide (anti-CCP)(+) patient samples with RF-derived peptides derived from RF(−)/anti-CCP(−) disease control samples, highlighting 13 upregulated peptides revealed by sparse partial least squares discriminant analysis (sPLS-DA). RF from 27 RF(+)/anti-CCP(+) rheumatoid arthritis (RA) patients, five RF(−)/anti-CCP(+) RA patients, 22 RF(−)/anti-CCP(−) RA patients, 28 RF(−)/anti-CCP(−) disease controls, and four RF(+)/anti-CCP(−) disease controls was isolated, digested into peptides, and analyzed using liquid chromatography tandem mass spectrometry (LC-MS/MS). Sequenced features (Ig-related and non-Ig-related features) were incorporated in the volcano plots with log2(fold change) on the x-axis and −log10(adjusted p-value) on y-axis. Shown are volcano plots comparing RF(+)/anti-CCP(+) patient samples with RF(−)/anti-CCP(−) control samples. Vertical dotted lines represent FC of 0.2 and 5, and horizontal dotted line represents an adjusted p-value cut-off <0.05. Thirteen features, annotated in purple on the volcano plot, situated in the framework or in the complementarity-determining regions, were more abundant in RF(+) RA than in controls, had an adjusted p-value <0.05, and were present after 50 repeats in sPLS-DA in cross-validation. In the right-hand panel, a heatmap is shown illustrating the median Z-score per peptide in each patient or disease control group, with peptides annotated per row.

Thirteen peptides situated on the variable region of immunoglobulins were considered discriminative peptides using sPLS-DA. These peptides were most abundant in the RA patient group, were present in more than half of the sPLS-DA repeats, and had an FC > 5 and an adjusted p-value <0.05. **Figure 8** illustrates the peptides on a volcano plot with a matched grouped median heatmap for the Z-score of these features. Interestingly, three of these peptides were also cross-validated using sPLS-DA in the proof-of-concept experiment (QVQLVESGGGLVK, PGQAPRLL, and SLSPGERATL).

A separate analysis was conducted on all features with MS/MS spectra from the main experiment, including all unidentified features. This independent analysis, with its own multiple testing correction, identified 343 upregulated features that were unsequenced and remained unidentified when using an adjusted p-value <0.05 and an FC > 5. Matching the characteristics of these upregulated unsequenced features with the characteristics of upregulated features in the proof-of-concept experiment (based on a mass/charge window < ± 0.01, retention time window < ± 1 min, same charge, and the presence of MS/MS scans) revealed 63 matching features. These features could, however, not be *de novo* sequenced, despite being analyzed in two separate experiments, being found in multiple samples, and furthermore having adequate MS/MS spectra. Some of these features were also present in selected RF(−)/anti-CCP(−) RA patients.

#### RF isotypes in the main experiment

**Figure 9:**
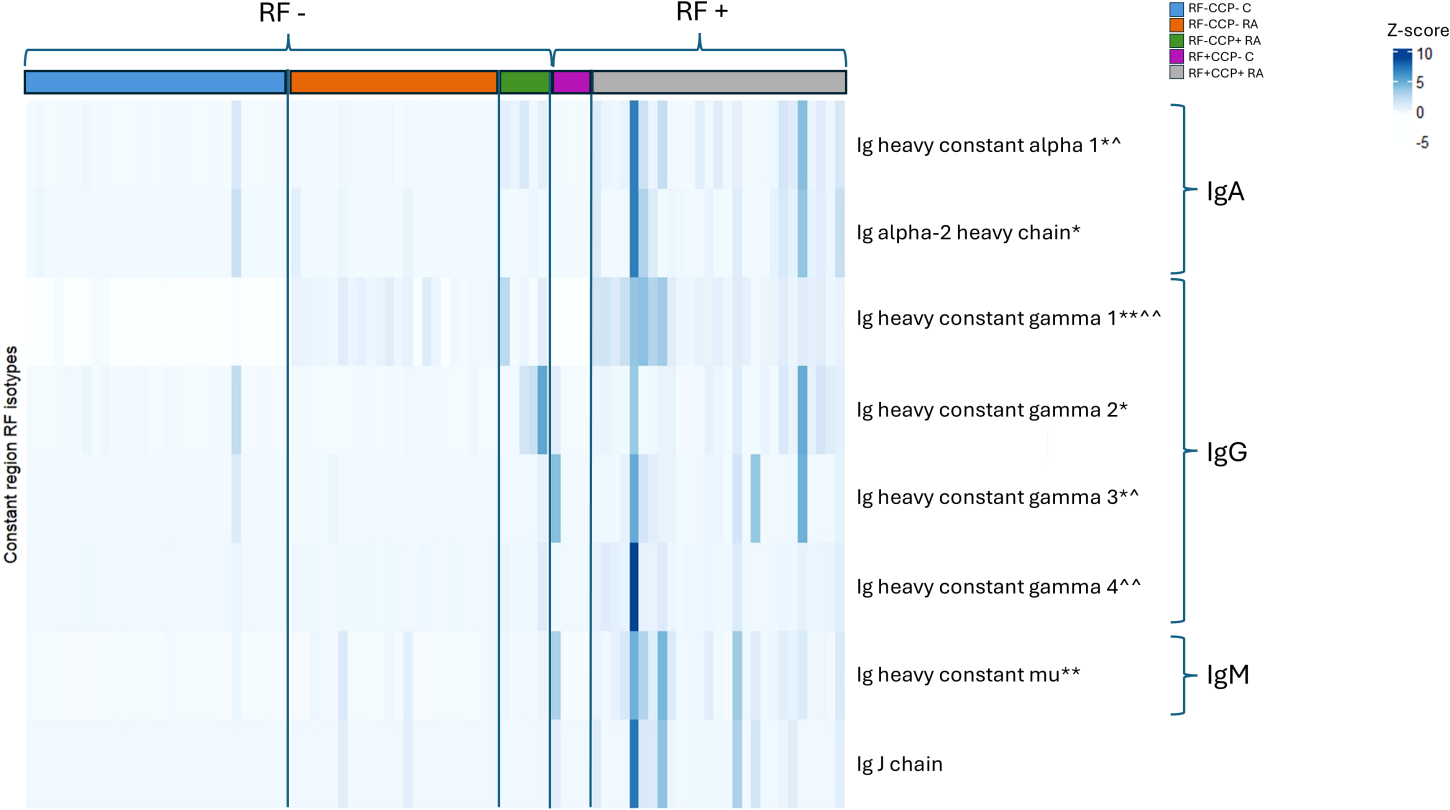
Heatmap representing Z-scores of different rheumatoid factor (RF) isotypes in rheumatoid arthritis (RA) patients and controls. RF from 27 RF(+)/anti-cyclic citrullinated peptide (anti-CCP)(+) RA patients, five RF(−)/anti-CCP(+) RA patients, 22 RF(−)/anti-CCP(−) RA patients, 28 RF(−) disease controls, and four RF(+) disease controls was isolated, digested into peptides, and analyzed using liquid chromatography tandem mass spectrometry (LC-MS/MS). Z-score of Ig heavy chain protein isotypes was compared in different disease groups. p-Value annotation for comparison of seropositive patient samples [RF(+)/anti-CCP(+)RA] versus disease control samples [RF(−)/anti-CCP(−) C] using Kruskal–Wallis testing followed by *post-hoc* Dunn’s test: *p < 0.05 and **p < 0.001. p-Value annotation for comparison of seronegative patient samples [RF(−)/anti-CCP(−) RA] versus disease control samples [RF(−)/anti-CCP(−) C] using Kruskal–Wallis testing followed by *post-hoc* Dunn’s test: ^p < 0.05 and ^^p < 0.001. Regarding IgM and IgG1, two UniProt accessions were found in PEAKS software (P0DOX5 and P01857 for IgM and P0DOX6 and P01871 for IgG1). As they have a high correlation and similar sequences, as shown in **Supplementary Figure 13**, only one accession number is illustrated in this figure. UniProt accession numbers corresponding with the protein description as listed in this figure are P01876, P0DOX2, P01857, P01859, P01860, P01861, and P01871.

**Figure 10:**
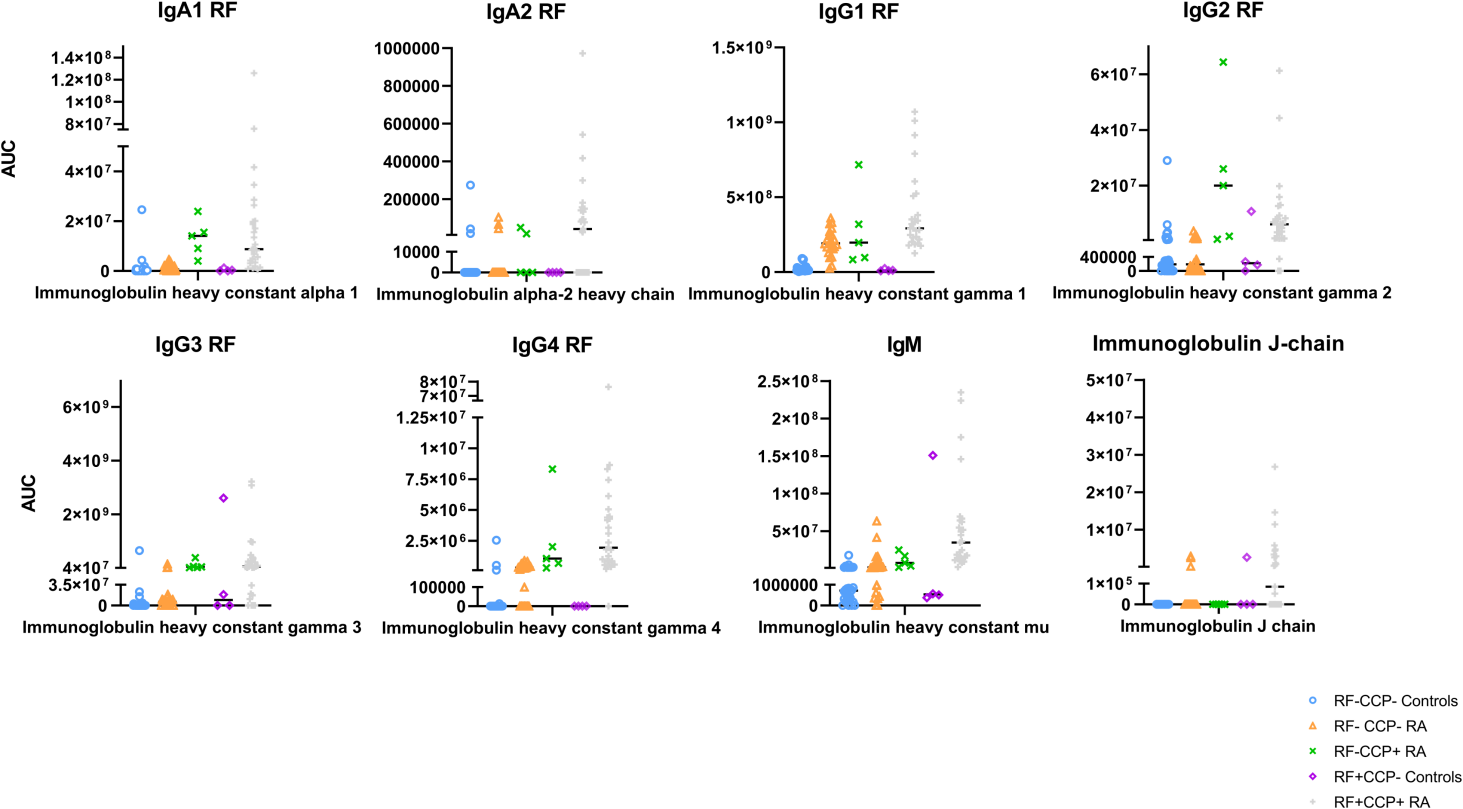
Quantification of Ig heavy chain rheumatoid factor (RF) isotypes in rheumatoid arthritis (RA) patients and controls. RF from 27 RF(+)/anti-cyclic citrullinated peptide (anti-CCP)(+) RA patients, five RF(−)/anti-CCP(+) RA patients, 22 RF(−)/anti-CCP(−) RA patients, 28 RF(−) disease controls, and four RF(+)/anti-CCP(−) disease controls was isolated, digested into peptides, and analyzed using liquid chromatography tandem mass spectrometry (LC-MS/MS). Protein abundance of Ig heavy chain isotype was obtained by totaling the peptide abundances of unique peptides per protein. Area under the curve for each protein is listed. Regarding IgM and IgG1, two UniProt accessions were found in PEAKS software (P0DOX5 and P01857 for IgM and P0DOX6 and P01871 for IgG1). As they have a high sequence similarity (shown in **Supplementary Figure 13**), only one accession number is illustrated in this figure. UniProt accession number P01871 represents the heavy mu chain, and accession P01857 represents IgG1 subclass.

**Figure 11:**
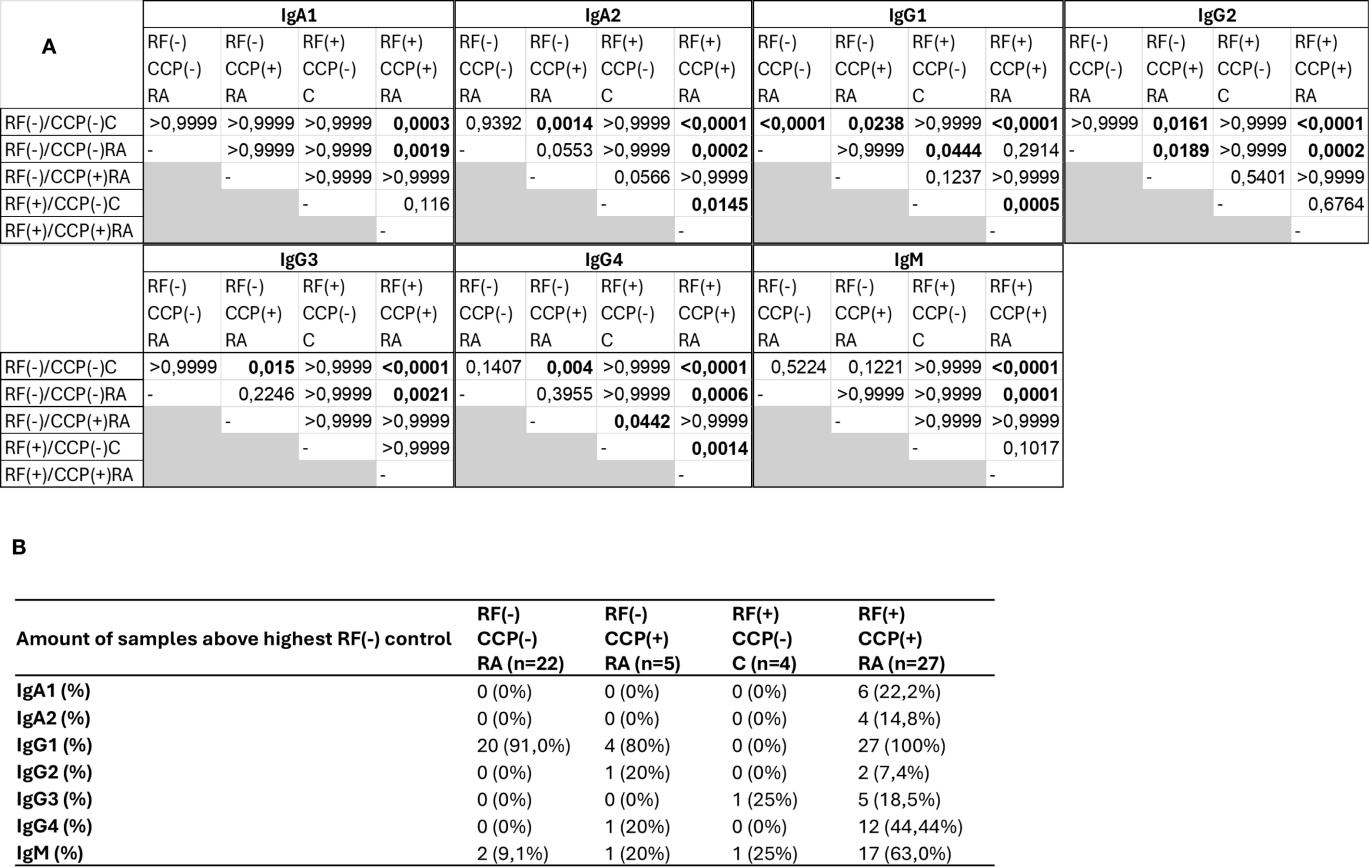
Summary statistics and analysis of data presented in **Figure 10**. Rheumatoid factor (RF) from 27 RF(+)/anti-cyclic citrullinated peptide (anti-CCP)(+) rheumatoid arthritis (RA) patients, five RF(−)/anti-CCP(+) RA patients, 22 RF(−)/anti-CCP(−) RA patients, 28 RF(−)/anti-CCP(−) disease controls, and four RF(+)/anti-CCP(−) disease controls was isolated, digested into peptides, and analyzed using liquid chromatography tandem mass spectrometry (LC-MS/MS). Data shown are the immunoglobulin isotype hits, quantified using unique peptides as relative protein quantification method. **(A)** p-Values presented in diagonal format matrices per RF isotype. Different groups were compared using Kruskal–Wallis statistical testing followed by a *post-hoc* Dunn’s test. **(B)** Number of samples considered positive with cut-off values based on the highest abundant sample in the RF(−)/anti-CCP(−) disease control group. Two UniProt accessions were found for the heavy constant γ1 chain, namely, P01857 and P0DOX5, situated in the same protein group. These are largely the same sequence and only marginally different (illustrated in **Supplementary Figure 13**).

A Z-score heatmap illustrates the abundance per isotype in **Figure 9**. The area under the curve (AUC) of the isotypes, a measure for relative protein quantification, is illustrated in **Figure 10**. For all isotypes, a significantly higher abundance was found in the RF(+)/anti-CCP(+) RA group compared to the RF(−)/anti-CCP(−) control group. For all isotypes, except IgG1, a significantly higher abundance was found in the RF(+)/anti-CCP(+) RA group compared to the RF(−)/anti-CCP(−) RA group. There was no statistical difference in abundance between RF(+)/anti-CCP(+) RA and RF(−)/anti-CCP(+) RA for all isotypes. For the IgM isotype, no significant difference was observed between the RF(+)/anti-CCP(+) RA group and the RF(+)/anti-CCP(−) control group. The abundance of all four IgG isotypes, but not of the IgM isotype, was higher in the RF(−)/anti-CCP(+) RA group than in the RF(−)/anti-CCP(−) control group, and the abundance of IgG2 isotype was higher in the RF(−)/anti-CCP(+) RA group than in the RF(−)/anti-CCP(−) RA group. The statistical analysis of different abundances and AUCs is summarized in **Figure 11A**. An overview of the total number of unique peptides per protein is given in **Supplementary Table 7**. **Figure 11B** illustrates the number (and percentage) of samples that had an isotype level that exceeded the highest isotype level obtained in RF(−)/anti-CCP(−) controls.

## Discussion

In this study, we aimed to characterize RF. RF was isolated from RF(+) RA patients, RF(−) RA patients, and non-inflammatory/non-autoimmune controls; digested into peptides; and analyzed using LC-MS/MS. sPLS-DA of Ig-related peptides revealed that the RA groups clustered separately from the control groups. We identified framework region sequences that were enriched in seropositive RA patients in two independent experiments. We also identified several variable region-related peptides that were consistently enriched in seropositive RA patients. Some of these peptides were also identified in the seronegative RA group, but not in the control group. However, data on RF-specific CDR sequences were limited because of the technical limitations of MS-based *de novo* sequencing.

Our study revealed comprehensive information on the RF isotypes. We found non-IgM-RF isotypes in seronegative patients. This is consistent with earlier studies that showed that 5%–18% of seronegative RA patients were negative for IgM RF but positive for either IgA- or IgG-RF (26). On that same line, nephelometry in a clinical setting has a higher sensitivity compared to single isotype immunoassay RF testing (13). In our MS-based experiments, 9.1% of RF(−)/anti-CCP(−) RA patients were positive for IgM RF. A substantial number of RF(−)/anti-CCP(−) RA patients were weakly positive for IgG1 RF. A possible explanation for the increased IgG1-RF intensity is the potential interference by IgG1 Fc coated on the ELISA plate. While it is likely that the IgG1 constant region is detected by MS analysis, a comparable level of intensity would be expected in control samples, which was not observed. Falkenburg et al. described that the Fc epitope of RF differed between RA and Sjögren’s disease (27). In subsequent studies, the same group found that IgM-RFs from healthy donors, patients with RA, and patients with Sjögren’s disease target distinct regions on the IgG-Fc and that the epitope that was associated with RA was targeted by both IgM-RF and IgA-RF (28).

The IgA-RF repertoire seems to be mainly related to pathology-associated specificities, suggesting an important role of IgA-RF in the pathogenesis of RA (10, 28). We found IgA1-RF mainly in RF(+) RA, which is in line with the suggestion that IgA-RF may be more specific for RA and potentially involved in the pathogenesis of RA (28).Currently, there is a lack of publicly available protein sequence databases and libraries specifically addressing CDRs in various diseases. This gap in knowledge of the Ig repertoire hampers the accurate identification of CDR peptides. Without proper amino acid sequence identification, achieving robust, reproducible, and accurate quantitative measurements is challenging primarily due to the large variability and the presence of unknown post-translational modifications. At present, the only viable method for identifying CDR peptides is *de novo* sequencing. Over the past decade, *de novo* sequencing has undergone significant improvements. However, the identification of CDRs in polyclonal Igs through *de novo* sequencing is much more complicated than *de novo* sequencing of monoclonal Igs, mainly due to the lower abundance of disease-specific CDRs in polyclonal Igs.

Standard database search (without *de novo* sequencing) revealed 143 Ig variable region peptides that were upregulated in RF(+)/anti-CCP(+) RA patients in the main experiment. Of these, 22 peptides were upregulated in the proof-of-concept experiment. The low number of overlapping peptides is probably caused by the limited sample size of the proof-of-concept experiment. A separate independent validation study would be needed to confirm the findings of the main experiment, either at the proteomic level or through single-cell B-cell receptor sequencing.

A total of 63 features were upregulated in two experiments, but remained unsequenced and unidentified after a database search and *de novo* sequencing, even though adequate MS/MS scans were obtained. Of note, some of these upregulated features were dominantly found in seronegative RA patients. These peptides are highly suggestive of being CDR-related but cannot be used to set up a (reliable clinical) assay, as no amino acid sequence could be allocated.

A limitation of this study is the absence of samples from other RF(+) inflammatory diseases, specifically systemic lupus erythematosus or Sjögren’s disease. Future studies should apply the MS-based approach to evaluate potential differences in RF composition between RA and other RF(+) inflammatory diseases. Another limitation is the relatively small sample size of the initial experiment, which may obscure potential upregulated features due to the lower statistical power associated with smaller sample sizes.

To summarize, our findings suggest that the nature of RF autoantibodies is complex. Current clinical immunoassays suffer from low specificity and poor harmonization and do not grasp this complexity (12). New techniques and approaches are on the horizon and may create opportunities to better characterize RF (at the Fab and isotype levels) and improve RF testing. Further advancements in MS-based analysis and software for *de novo* sequencing of the variable Ig region will allow better, clinically useful RF characterization.

## Data Availability

The mass spectrometry proteomics data have been deposited to the ProteomeXchange Consortium via the PRIDE partner repository with the dataset identifier PXD063283 and 10.6019/PXD063283.
