# Supplementary material for: Mass spectrometry-based analysis of rheumatoid factor
Source: Front Immunol. 2025 Oct 10;16:1644334. doi: 10.3389/fimmu.2025.1644334 (PMC12549570; doi:10.3389/fimmu.2025.1644334)
Supplement: Supplementary file 1 [file DataSheet1.pdf]

	Total (n=11)	RF(-)/anti-CCP(-)C (n=4)	RF(-)/anti-CCP(-)RA (n=3)	RF(+)/anti-CCP(+)RA (n=4)
Male, %	18,2	0	33,3	25
Female, %	81,8	100	66,7	75
Age (years) , mean±SD	52,8±11,3	44,0±13,3	53,0±3,7	61,5±2,9
Ever Smoking, %	18,2	0	66,7	0
PY, mean±SD	16,5±3,5	0	16,5±3,5	0
DAS28-ESR, mean±SD*	5,9±1,1	N/A	6,5±1,0	5,4±0,8
DAS28-CRP, mean±SD*	5,2±1,4	N/A	6,2±1,3	4,4±0,8
RF, mean±SD°	67,1±97,1	0,0±0,0	0,0±0,0	184,5±65,3
anti-CCP, mean±SD^	94,6±150,3	0,7±0,2	1,3±0,9	258,5±141,2

* Only recorded in RA patients
° Values <20 U/ml are considered 0 due to no routine measurement <20 U/ml
^ Values >340 IU/ml are considered 340 IU/ml due to no routine measurement >340 UI/ml
Normal value RF: <40U/ml
Normal value ACPA: <7 IU/ml

Supplemental Table 1: Baseline characteristics from samples used in the first experiment with sera from 4 RF(+)/anti-CCP(+) RA patients, 3 RF(-)/anti-CCP(-) RA patients and 4 RF(-)/anti-CCP(-) controls. SD = standard deviation, PY = pack years.

	Total (n=86)	RF(-)/anti-CCP(-)C (n=28)	RF(+)/anti-CCP(-)C (n=4)	RF(-)/anti-CCP(-)RA (n=22)	RF(+)/anti-CCP(+)RA (n=27)	RF(-)/anti-CCP(+)RA (n=5)
Male, %	22,1	3,6	25,0	36,4	33,3	0,0
Female, %	77,9	96,4	75,0	63,6	66,7	100,0
Age (years) , mean±SD	49,6±14,8	43,1±14,5	55,8±11,5	54,5±13,7	51,7±13,9	49,0±13,6
Ever Smoking, %	37,2	21,4	25,0	45,5	48,1	40,0
PY, mean±SD	18,3±14,3	13,8±12,1	33±0	15,3±8,3	22,7±17,5	10,5±9,5
DAS28-ESR, mean±SD*	5,6±1,1	N/A	N/A	6,0±1,1	5,3±1,1	5,0±0,7
DAS28-CRP, mean±SD*	5,0±1,1	N/A	N/A	5,6±1,0	4,7±1,0	4,7±0,9
RF (U/ml), mean±SD°	147,7±278,7	0,0	249,5±208,5	0,0	428,5±342,8	18,2±14,9
anti-CCP (IU/ml), mean±SD^	102,9±149,0	0,0	0,0	1,0±1,1	278,1±105,7	263,7±128,5

* Only recorded in RA patients
° Values <20U/ml are considered 0 due to no routine measurement <20U/ml
^ Values >340 IU/ml are considered 340 IU/ml due to no routine measurement >340 UI/ml
Normal value RF: <40U/ml
Normal value ACPA <7 IU/ml

Supplemental Table 2: baseline characteristics of patient samples included in the second experiment with sera from 27 RF(+)/anti-CCP(+) RA patients, 5 RF(-)/anti-CCP(+) RA patients, 22 RF(-)/anti-CCP(-) RA patients, 28 RF(-)/anti-CCP(-) disease controls (C), 4 RF(+)/anti-CCP(-) disease controls (C). SD = standard deviation, PY = pack years.

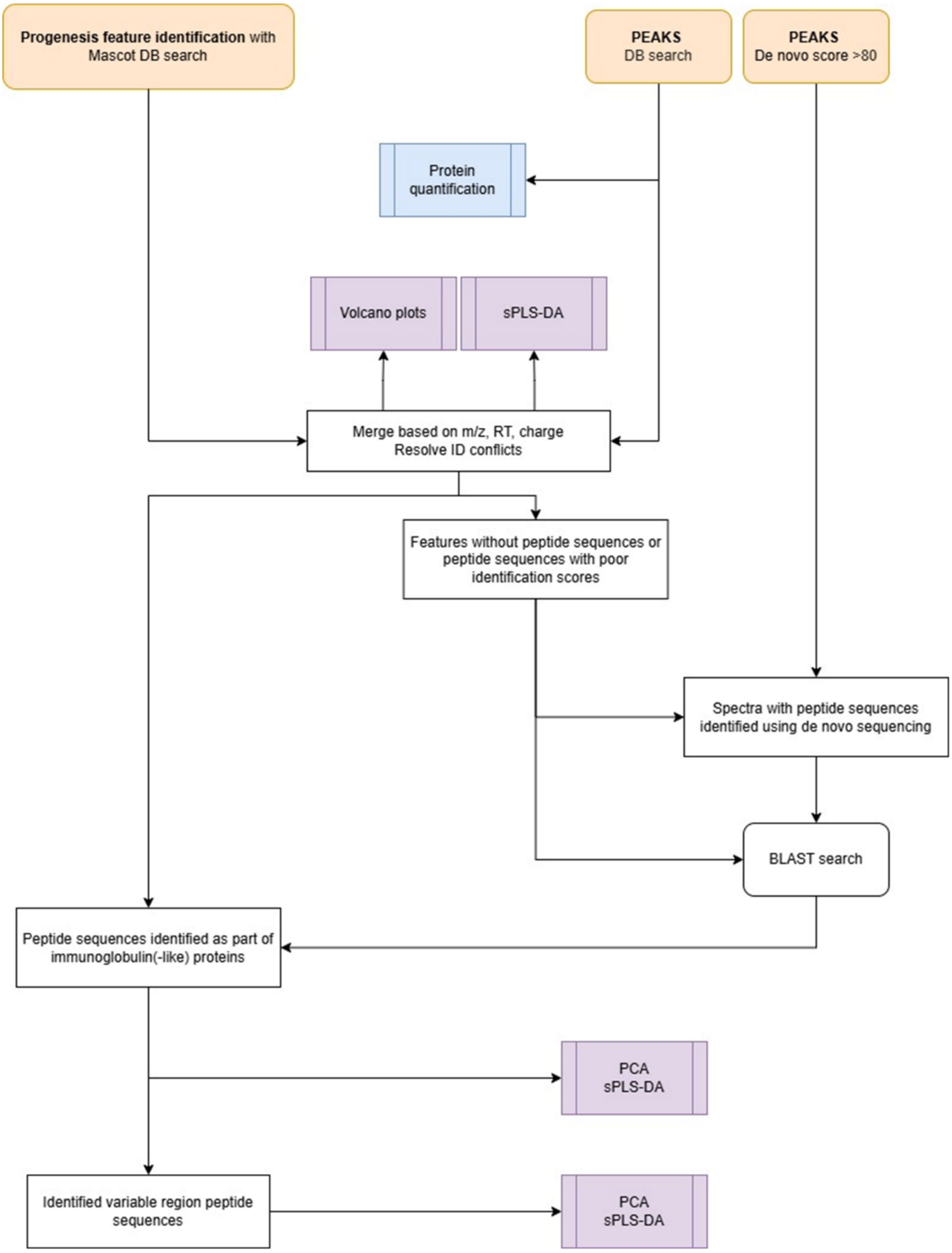

Supplemental Figure 1: Bioinformatic overview

Overview of the bioinformatics analysis and statistical analysis starting from the output raw data files from the LC-MS/MS analysis. BLAST search was performed using IgBlast and IMGT according to specifications described in Materials and Methods. DB = database. RT= retention time. ID: identification.

Parent Mass Error Tolerance: 10.0 ppm
Fragment Mass Error Tolerance: 0.02 Da
Precursor Mass Search Type: monoisotopic
Enzyme: Specified by each sample
Max Missed Cleavages: 2
Peptide Length Range: 6 - 45
Fixed Modifications:
  Carbamidomethylation (+57.02)
Variable Modifications:
  Deamidation (NQ) (+0.98)
  Oxidation (M) (+15.99)
Max Variable PTM Per Peptide: 2

Supplemental Table 3: Overview database search parameters for both experiments.

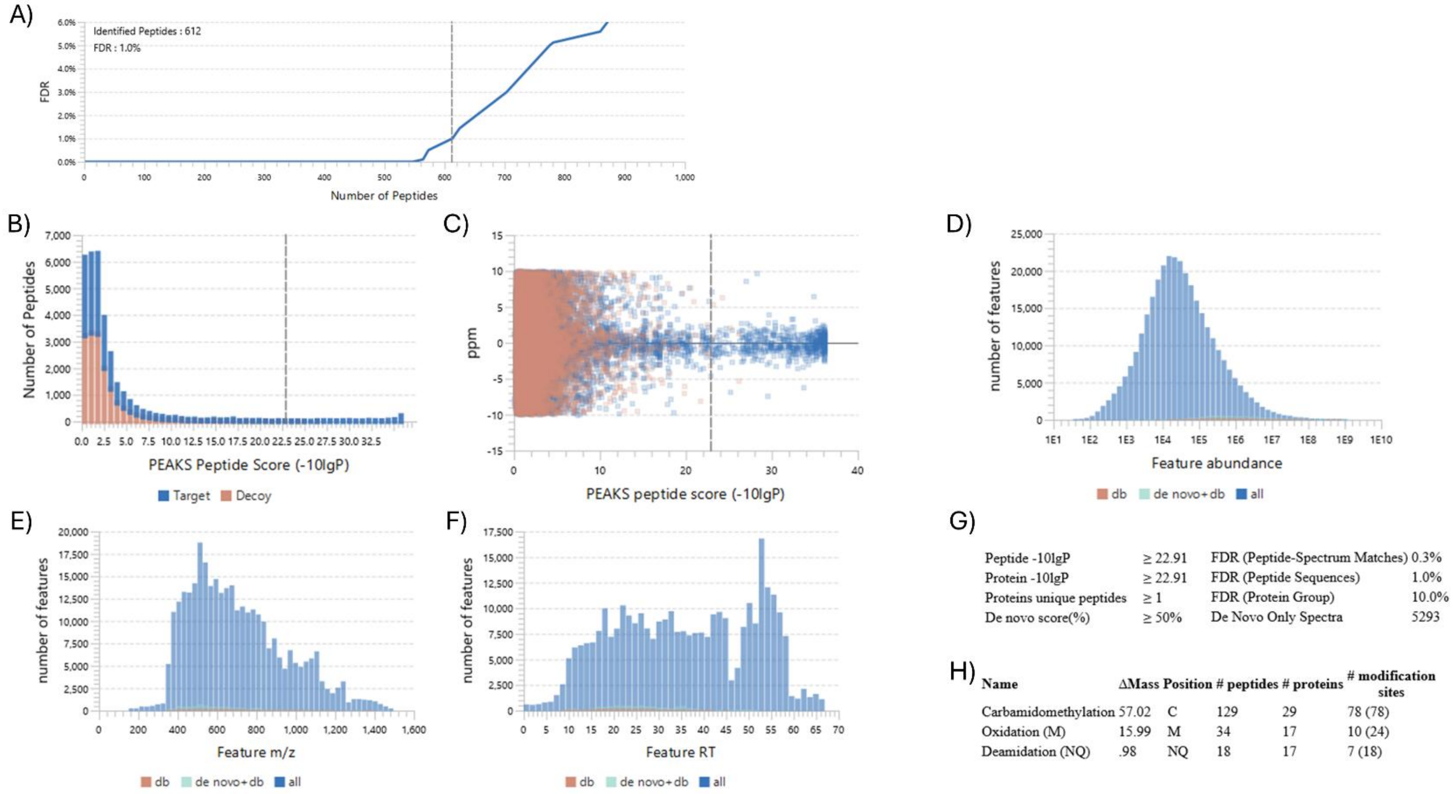

Supplemental Figure 2: Result statistics overview of the proof-of-concept experiment, , illustrated using PEAKS software.

Data statistics overview of feature identification hits using PEAKS. A) FDR curve of identified peptides. On the X-axis, the number of peptides is shown and on the Y-axis the corresponding FDR. Vertical line represents a peptide FDR 1%. B) A stacked histogram illustrating score distribution of PEAKS peptide score of target and decoy matches in each score interval. The vertical line indicates the score threshold used in this experiment. C) A scatterplot showing the peptide score distribution versus precursor mass error in ppm for all peptide spectrum matches. The vertical line indicates the score threshold used in this experiment. D) Distribution of identified peptide features per abundance. E) Distribution of m/z peptide feature detection. F) Distribution of RT peptide features detection. G) Result filtration parameters and statistics of filtered results. H) Post translational modification profiling of all identified peptides. m/z = mass to charge, RT = retention time

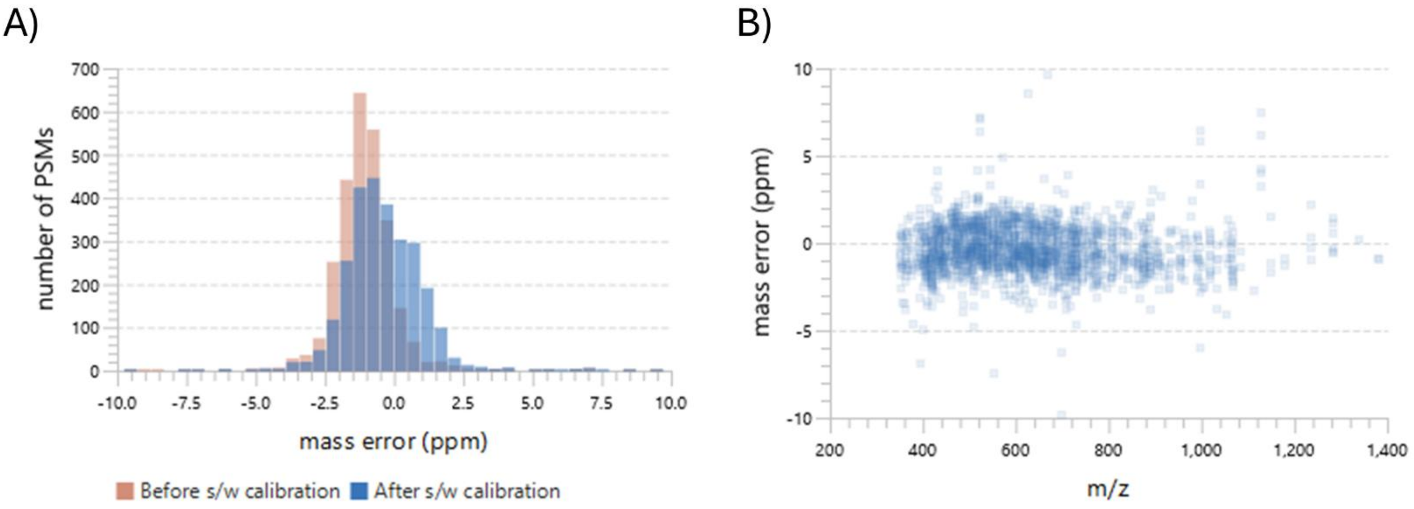

Supplemental Figure 3: precursor mass error of peptide-spectrum matches (PSM) in the proof-of-concept experiment, illustrated by PEAKS software.

Data shown are the data statistics overview of feature identification hits using PEAKS. A) distribution of precursor mass error in ppm. B) Scatterplot of precursor m/z versus precursor mass error in ppm. m/z = mass to charge.

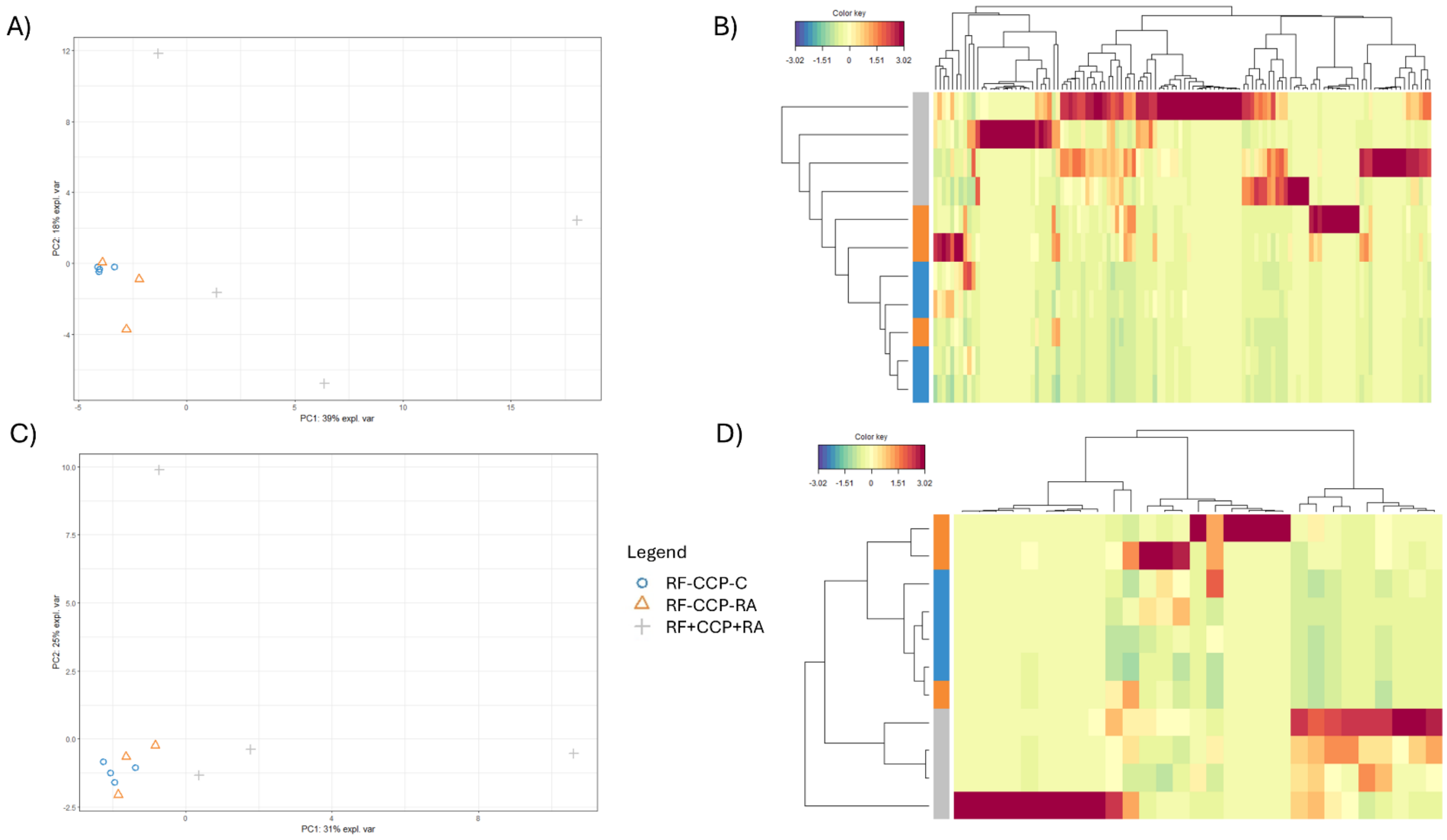

Supplemental Figure 4: Principal component analysis (PCA) after RF isolation

RF from 4 RF(+)/anti-CCP(+) RA patients, 3 RF(-)/anti-CCP(-) RA patients and 4 RF(-)/anti-CCP(-) disease controls was isolated, digested into peptides and analyzed by LC-MS/MS. A) PCA of all Ig related peptides in RF(+)/anti-CCP(+) RA patients, RF(-)/anti-CCP(-) RA patients and RF(-)/anti-CCP(-) disease control patient samples. B) Heatmap after hierarchical clustering of the Ig related features responsible for the variance of the PCA. Heatmap color key indicates the difference in Z-score (the standardized, normalized abundance). C) PCA plotting the variance in variable region related peptides in RF(+)/anti-CCP(+) RA patients, RF(-)/anti-CCP(-) RA patients and RF(-)/anti-CCP(-) disease control patient samples. D) Heatmap after hierarchical clustering of the variable region peptides responsible for the variance of the PCA. Heatmap color key indicates the difference in Z-score.

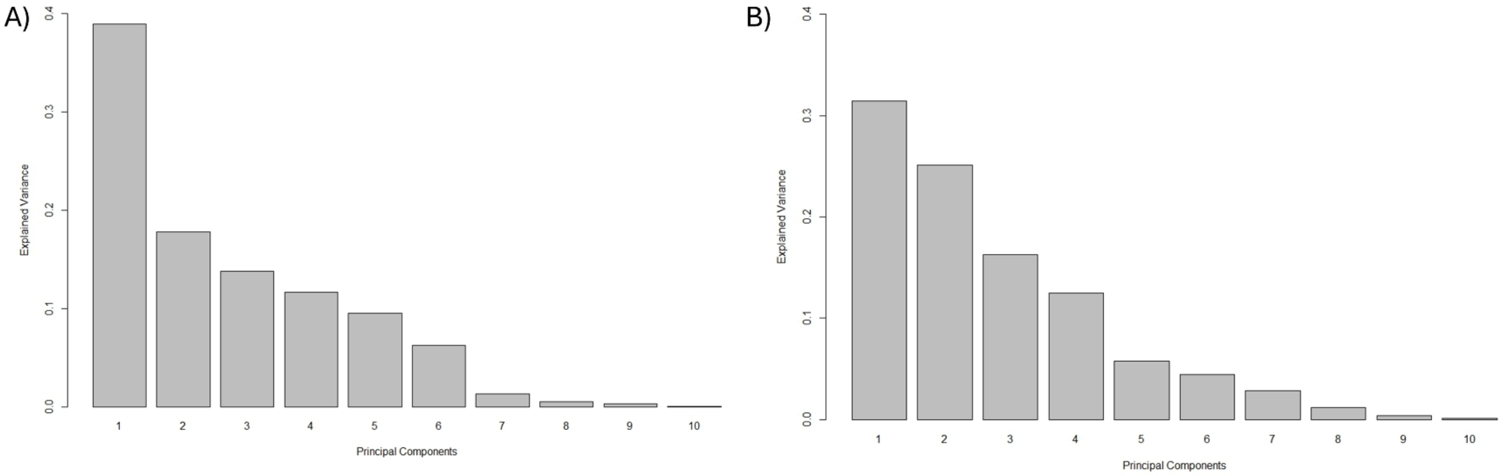

Supplemental Figure 5: Elbow plot illustrating the cumulative variance explained by principal components.

RF from 4 RF(+)/anti-CCP(+) RA patients, 3 RF(-)/anti-CCP(-) RA patients and 4 RF(-)/anti-CCP(-) disease controls was isolated, digested into peptides and analyzed by LC-MS/MS. PCA was used to evaluate variance within the samples. The figures are elbow plots illustrating the proportion of total variance accounted for by increasing numbers of principal components.

A) Elbow plot; explained variance in principal components used for plotting PCA regarding all immunoglobulin related peptides.

B) Elbow plot; explained variance in principal components used for plotting PCA regarding all variable region peptides.

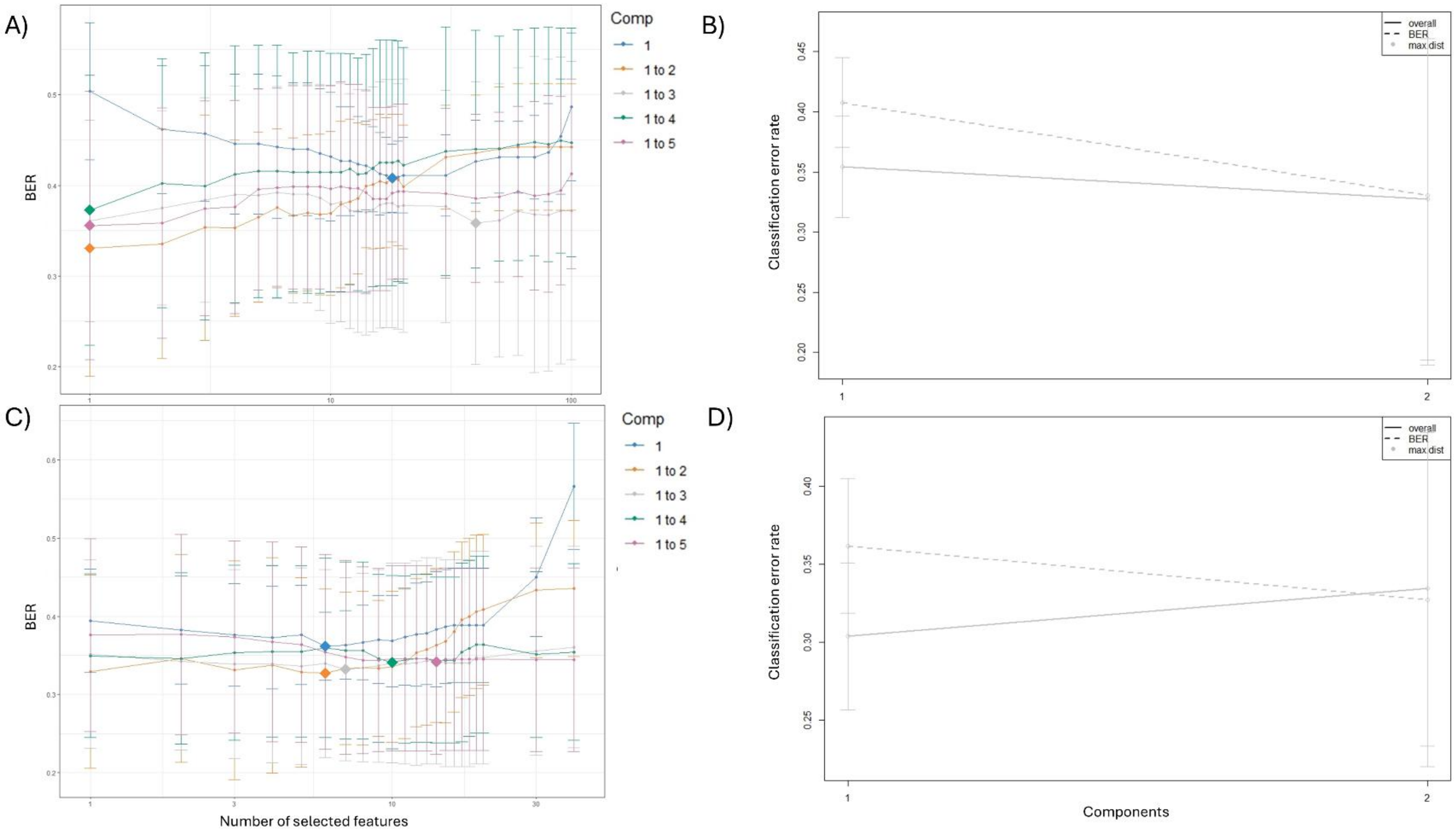

Supplemental Figure 6: Classification error rates and balanced error rates (BER) for the PLS-DA models used.

RF from 4 RF(+)/anti-CCP(+) RA patients, 3 RF(-)/anti-CCP(-) RA patients and 4 RF(-)/anti-CCP(-) disease controls was isolated, digested into peptides and analyzed by LC-MS/MS. sPLS-DA was used to evaluate differentiating peptides. The plots illustrate the overall classification error rates and BER obtained from cross-validation of the PLS-DA model. The classification error rate represents the proportion of misclassified samples across all classes, while the BER accounts for class imbalance by averaging the misclassification error for each class equally.

A) BER and number of selected features per component after sPLS-DA analysis including all immunoglobulin related peptides.

B) Classification error rate per component after sPLS-DA analysis including all immunoglobulin related peptides

C) BER and number of selected features per component after sPLS-DA analysis including all variable region peptides.

D) Classification error rate per component after sPLS-DA analysis including all variable region peptides.

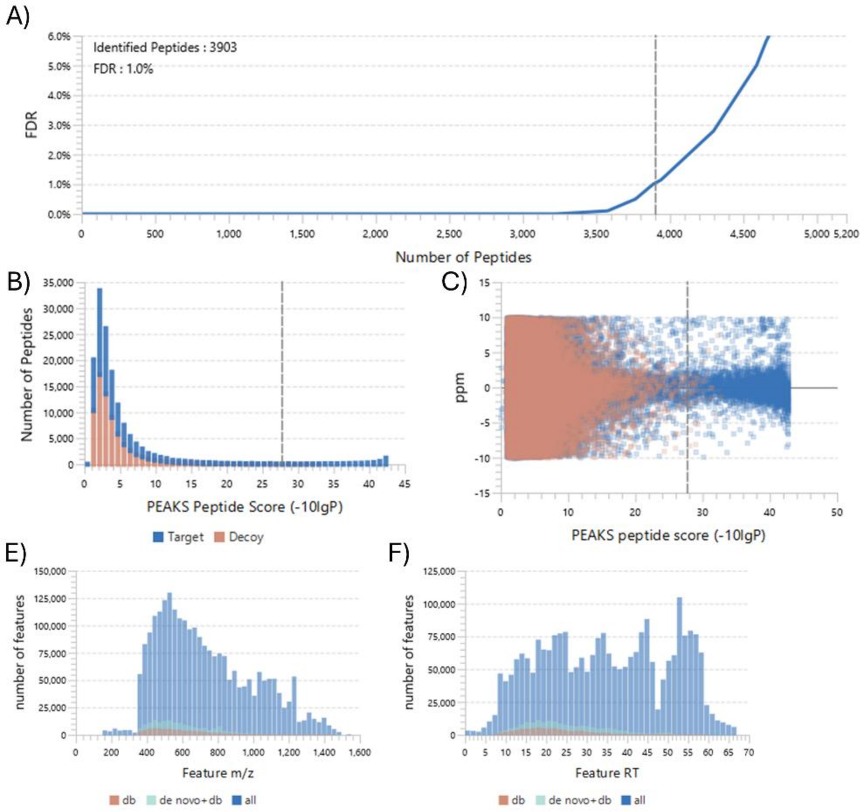

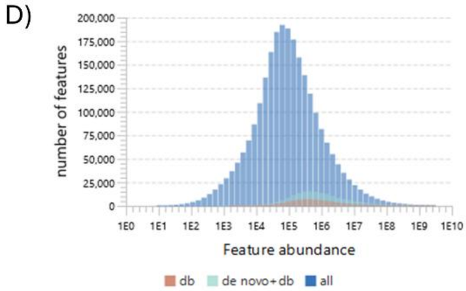

G)

Peptide -10lgP	≥ 27.76	FDR (Peptide-Spectrum Matches)	0.4%
Protein -10lgP	≥ 15	FDR (Peptide Sequences)	1.0%
Proteins unique peptides	≥ 1	FDR (Protein Group)	7.3%
De novo score(%)	≥ 50%	De Novo Only Spectra	139957

H)

Name	ΔMass	Position	# peptides	# proteins	# modification sites
Carbamidomethylation	57.02	C	575	104	446 (446)
Deamidation (NQ)	.98	NQ	252	109	40 (258)
Oxidation (M)	15.99	M	239	60	105 (156)

Supplemental Figure 7: Result statistics overview of the main experiment, illustrated by PEAKS software.

Data statistics overview of feature identification hits using PEAKS. A) FDR curve of identified peptides. On the X-axis, the number of peptides is shown and on the Y-axis the corresponding FDR. Vertical line represents a peptide FDR 1%. B) A stacked histogram illustrating score distribution of PEAKS peptide score of target and decoy matches in each score interval. The vertical line indicates the score threshold used in this experiment. C) A scatterplot showing the peptide score distribution versus precursor mass error in ppm for all peptide spectrum matches. The vertical line indicates the score threshold used in this experiment. D) Distribution of identified peptide features per abundance. E) Distribution of m/z peptide feature detection. F) Distribution of RT peptide features detection. G) Result filtration parameters and statistics of filtered results. H) Post translational modification profiling of all identified peptides. m/z = mass to charge, RT = retention time.

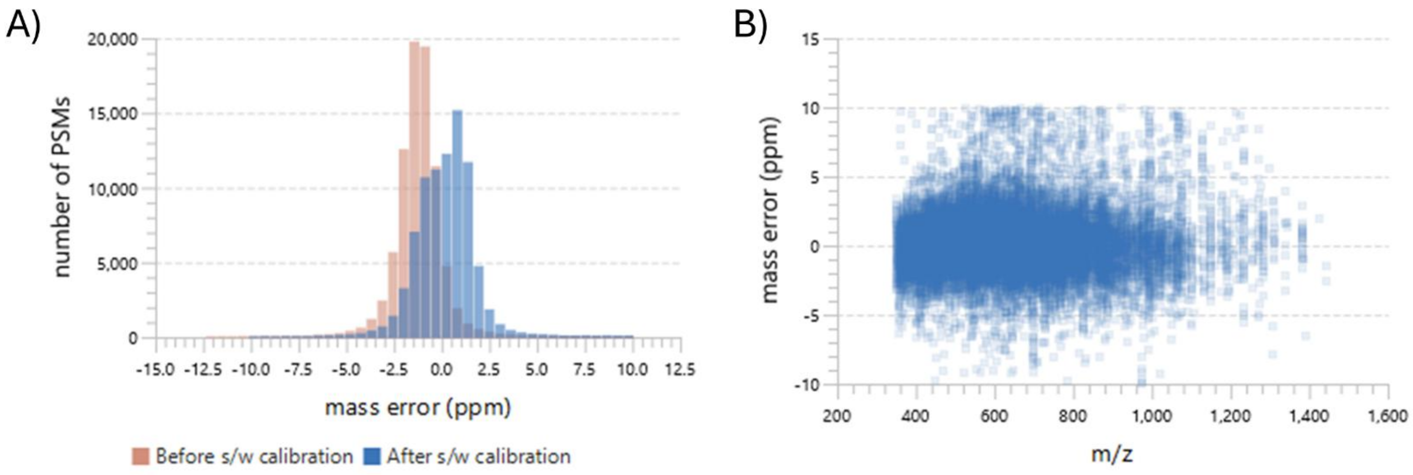

Supplemental Figure 8: precursor mass error of peptide-spectrum matches (PSM) in the main experiment, illustrated by PEAKS software. A) distribution of precursor mass error in ppm. B) Scatterplot of precursor m/z versus precursor mass error in ppm.

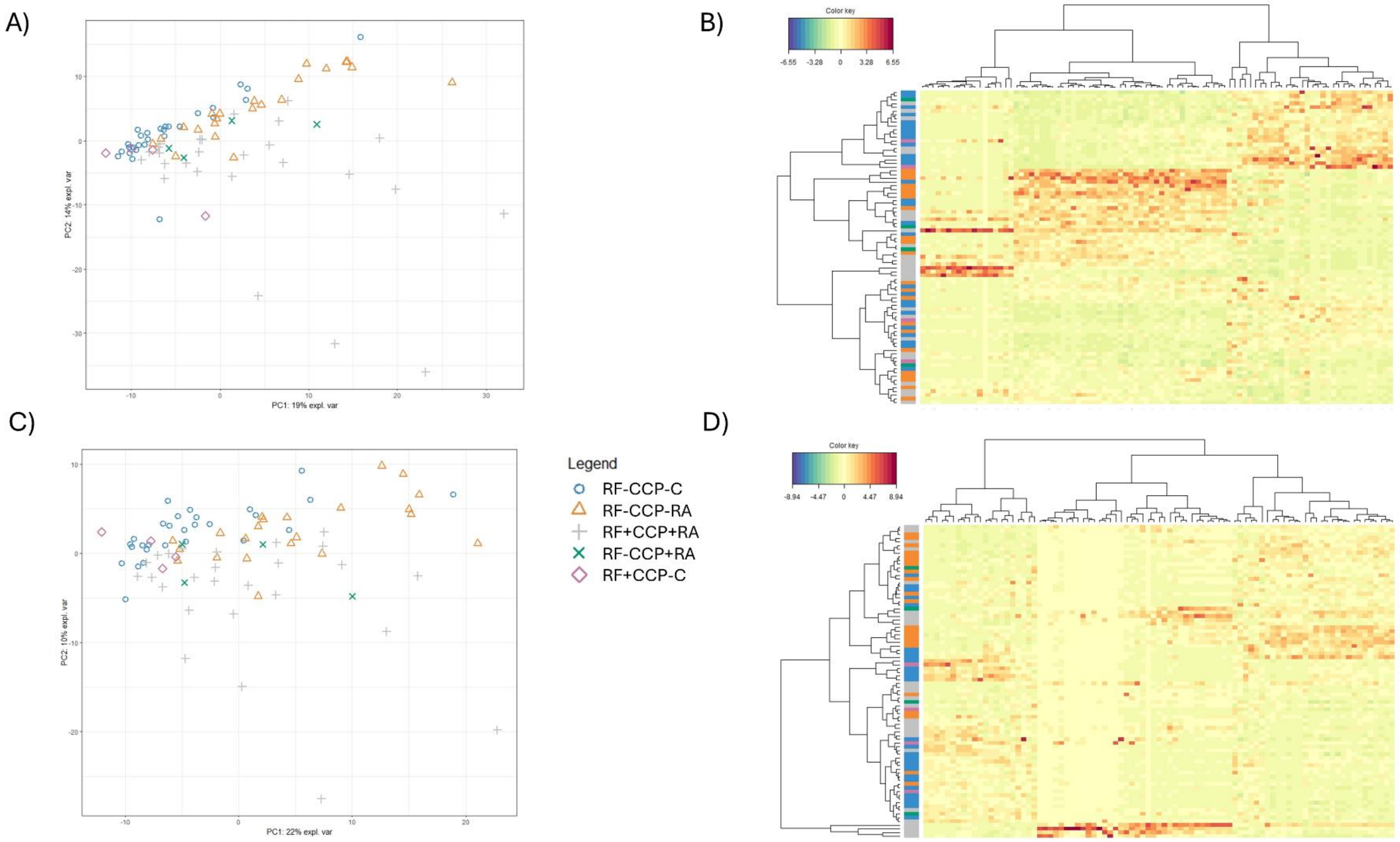

Supplemental Figure 9: PCA after RF isolation

RF from 27 RF(+)/anti-CCP(+) RA patients, 5 RF(-)/anti-CCP(+) RA patients, 22 RF(-)/anti-CCP(-) RA patients, 28 RF(-) disease controls and 4 RF(+) disease controls was isolated, digested into peptides and analyzed by LC-MS/MS. A) PCA plotting the variance of Ig related peptides in sera from different groups. B) Heatmap after hierarchical clustering of the Ig related peptides responsible for the variance of the PCA. Heatmap color key indicates the difference in Z-score. C) PCA plotting the variance in variable region related peptides in sera from different groups. D) Heatmap after hierarchical clustering of the variable region peptides responsible for the variance of the PCA. Heatmap color key indicates the difference in Z-score.

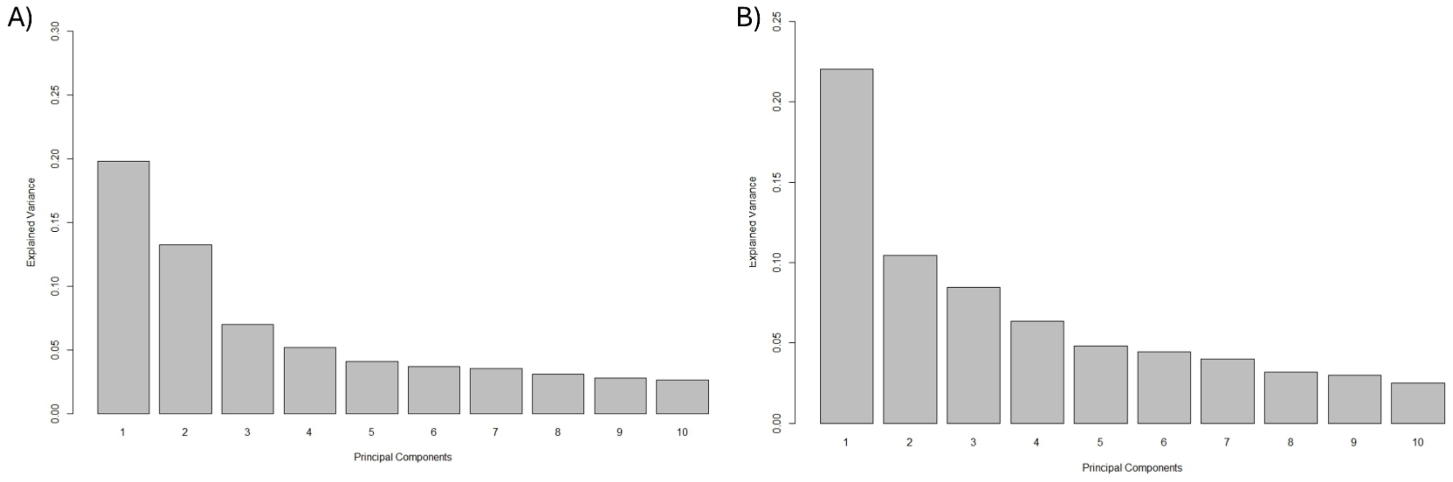

Supplemental Figure 10: Elbow plot illustrating the cumulative variance explained by principal components.

RF from 27 RF(+)/anti-CCP(+) RA patients, 5 RF(-)/anti-CCP(+) RA patients, 22 RF(-)/anti-CCP(-) RA patients, 28 RF(-) disease controls and 4 RF(+) disease controls was isolated, digested into peptides and analysed by LC-MS/MS. sPLS-DA was used to evaluate differentiating peptides. PCA was used to evaluate variance within the samples. The figures are elbow plots illustrating the proportion of total variance accounted for by increasing numbers of principal components (PCs).

A) Elbow plot; explained variance in principal components used for plotting PCA regarding all immunoglobulin related peptides.

B) Elbow plot; explained variance in principal components used for plotting PCA regarding all variable region peptides.

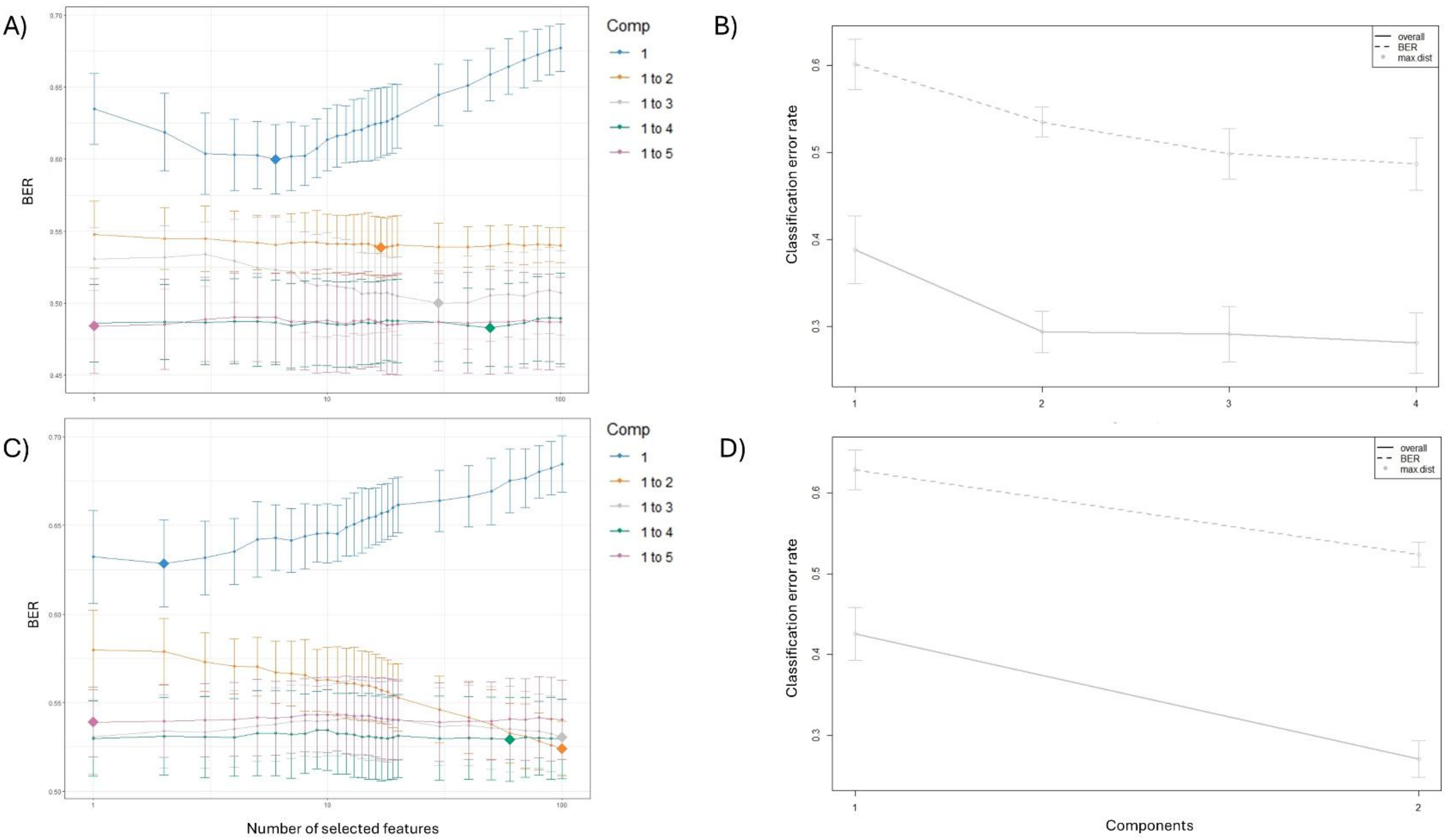

Supplemental Figure 11: Balanced error rate (BER) and classification error rates used in the sPLS-DA models.

RF from 27 RF(+)/anti-CCP(+) RA patients, 5 RF(-)/anti-CCP(+) RA patients, 22 RF(-)/anti-CCP(-) RA patients, 28 RF(-) disease controls and 4 RF(+) disease controls was isolated, digested into peptides and analysed by LC-MS/MS. sPLS-DA was used to evaluate differentiating peptides. These plots illustrates the overall classification error rates and balanced error rates (BER) obtained from cross-validation of the PLS-DA model. The classification error rate represents the proportion of misclassified samples across all classes, while the BER accounts for class imbalance by averaging the misclassification error for each class equally.

A) Balanced error rate and number of selected features per component after sPLS-DA analysis including all immunoglobulin related peptides.

B) Classification error rate per component after sPLS-DA analysis including all immunoglobulin related peptides

C) Balanced error rate and number of selected features per component after sPLS-DA analysis including all variable region peptides.

D) Classification error rate per component after sPLS-DA analysis including all variable region peptides.

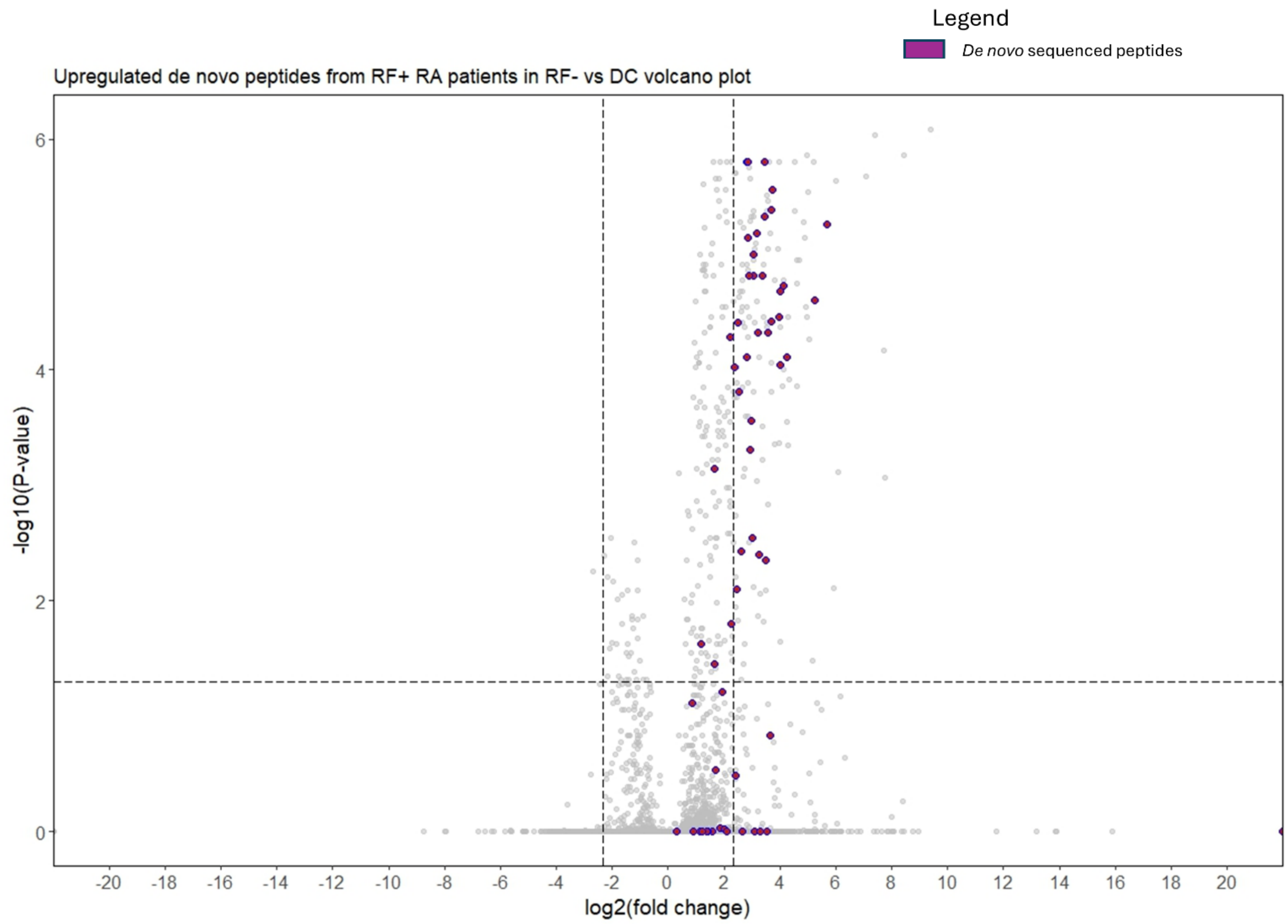

Supplemental Figure 12: Volcano plot comparing RF(-)/anti-CCP(-) patient (n=22) samples with RF(-)/anti-CCP(-) disease control (n=28) samples.

RF from 27 RF(+)/anti-CCP(+) RA patients, 5 RF(-)/anti-CCP(+) RA patients, 22 RF(-)/anti-CCP(-) RA patients, 28 RF(-)/anti-CCP(-) disease controls and 4 RF(+)/anti-CCP(-) disease controls was isolated, digested into peptides and analyzed by LC-MS/MS. Data shown are for all sequenced features (immunoglobulin-related, non-immunoglobulin related and *de novo* sequenced peptides). Vertical dotted lines represent FC 0.2 and 5 and horizontal dotted line represents an adjusted p-value <0.05. Features in purple represent *de novo* sequenced peptides upregulated when comparing RF(+)/anti-CCP(+)RA patients with RF(-)/anti-CCP(-) disease controls. Thirty-seven sequences (from the 61 *de novo* sequenced peptides depicted) are upregulated in both the RF(-)/anti-CCP(-) RA group as in the RF(+)/anti-CCP(+) RA group.

m/z	RT	Charge	Peptide	*De novo* score	Adj P value	FC	Group
358,21	13,0	2	SPLRDK	84	1,50E-08	75,6	RF(+)
364,54	11,8	3	RRDQTLRF	82	3,73E-09	69,7	RF(+)
364,54	11,8	3	RRDQTLRF	82	9,16E-07	11,2	RF(-)
365,23	16,9	2	LEVQLK	87	2,53E-08	17,0	RF(+)
365,23	16,9	2	LEVQLK	87	5,62E-08	7,9	RF(-)
366,42	14,4	4	TVEDMPFEDHVK	82	6,71E-06	16,8	RF(+)
374,24	21,0	2	LQVLFK	96	6,71E-07	38,4	RF(+)
381,86	12,2	3	SGNTFRGYNK	81	9,53E-09	13,9	RF(+)
384,24	16,0	2	LPALPEK	82	2,44E-09	29,5	RF(+)
384,24	16,0	2	LPALPEK	82	8,28E-07	9,5	RF(-)
387,23	18,0	2	RLEEVK	90	1,43E-09	77,6	RF(+)
395,55	12,7	3	EALPVPVCTAK	84	6,76E-10	53,4	RF(+)
398,18	16,3	5	WKTMSSVMHEALHNHY	86	7,64E-06	9,0	RF(+)
405,70	14,2	2	NNFKCK	83	2,14E-08	16,6	RF(+)
412,79	13,0	5	SVADGYGSVMHEALHNGHY	80	6,06E-10	11,4	RF(+)
412,79	13,0	5	SVADGYGSVMHEALHNGHY	80	3,04E-09	8,3	RF(-)
424,57	16,8	3	GVALHHPNVAEK	98	3,89E-10	5,2	RF(-)
426,73	21,1	2	WGGLTRY	84	1,65E-06	23,2	RF(+)
427,72	15,2	2	KFGERAF	96	2,01E-06	9,3	RF(+)
431,89	21,7	3	TVLHQDAPLNW	86	4,35E-10	8,7	RF(+)
431,89	21,7	3	TVLHQDAPLNW	86	1,94E-08	5,2	RF(-)
438,23	21,3	3	VSKHQDWLNGK	82	1,16E-09	9,6	RF(+)
438,23	21,3	3	VSKHQDWLNGK	82	3,18E-08	5,8	RF(-)
442,90	17,2	3	TVLHQDAFPNGK	80	1,43E-08	8,0	RF(+)
442,90	18,1	3	TVLHQDAFPNGK	80	1,31E-06	5,1	RF(+)
444,23	16,7	3	TVLHQDTAFNGK	83	3,02E-08	54,4	RF(+)
444,23	16,7	3	TVLHQDTAFNGK	83	5,04E-09	38,1	RF(-)
448,23	17,7	3	TVLHQDPSFNGK	82	3,89E-10	8,0	RF(+)
448,23	17,7	3	TVLHQDPSFNGK	82	1,44E-09	7,2	RF(-)
448,23	15,5	3	TNRSDQYGRVF	82	6,32E-09	10,2	RF(+)
448,23	20,8	3	TVLHQDYAPNGK	81	1,61E-09	13,8	RF(+)
448,23	20,8	3	TVLHQDYAPNGK	81	1,02E-07	7,5	RF(-)
448,23	15,0	3	TNRSDQYGRVF	82	6,30E-09	12,0	RF(+)
448,23	15,0	3	TNRSDQYGRVF	82	7,70E-07	6,2	RF(-)
448,56	18,6	3	TVLHQDSPFDGK	80	3,12E-10	12,1	RF(+)
448,56	18,6	3	TVLHQDSPFDGK	80	9,37E-10	10,8	RF(-)
451,75	11,6	2	VAVLSNDGK	91	1,76E-09	37,9	RF(+)
451,75	11,6	2	VAVLSNDGK	91	1,56E-08	19,2	RF(-)
451,90	17,9	3	TVVNHGEWLGGGK	80	7,63E-08	7,3	RF(+)
456,57	20,9	3	TVLHGQDWLGGGK	94	8,41E-10	14,9	RF(+)
456,57	20,9	3	TVLHGQDWLGGGK	94	9,53E-09	9,4	RF(-)
457,24	20,6	3	TTGLHQDWLGGGK	83	9,26E-08	5,7	RF(+)
478,74	19,3	2	WGGGAYFAK	80	6,35E-10	22,9	RF(+)
478,74	19,3	2	WGGGAYFAK	80	1,84E-08	16,3	RF(-)
486,25	26,0	2	TTQVVHTW	80	3,12E-10	8,7	RF(+)
486,25	26,0	2	TTQVVHTW	80	3,12E-10	11,0	RF(-)

501,48	13,1	4	WKTMHEALHNHY	84	5,42E-10	7,6	RF(+)
501,48	13,1	4	WKTMHEALHNHY	84	7,76E-09	5,6	RF(-)
506,27	25,9	2	TVLHQDLW	86	3,89E-10	26,3	RF(+)
506,27	25,9	2	TVLHQDLW	86	4,17E-09	16,2	RF(-)
511,74	16,2	4	WGYRSSVMHEALHNGHY	87	3,35E-06	8,2	RF(+)
539,77	38,4	2	FDTLLEPPF	84	1,25E-08	10,5	RF(+)
539,77	38,4	2	FDTLLEPPF	84	8,07E-10	12,9	RF(-)
552,26	26,9	2	WYVDRTTY	84	2,91E-06	19,2	RF(+)
553,30	23,9	2	APVNLLTVMF	83	2,11E-07	Inf	RF(+)
576,28	14,8	2	SSGSPLYSPEK	98	3,12E-10	6,2	RF(+)
576,28	14,8	2	SSGSPLYSPEK	98	3,12E-10	7,3	RF(-)
580,29	29,9	2	SVNLWSPAVW	80	3,12E-10	6,0	RF(+)
580,29	29,9	2	SVNLWSPAVW	80	3,12E-10	7,1	RF(-)
597,95	24,9	3	QQTEPGSSGSPLYSPEK	90	6,39E-07	7,6	RF(+)
597,95	24,9	3	QQTEPGSSGSPLYSPEK	90	7,66E-09	13,0	RF(-)
597,95	25,2	3	QQTEPGSSGSPLYSPEK	90	1,80E-06	5,1	RF(+)
597,95	25,2	3	QQTEPGSSGSPLYSPEK	90	1,65E-06	5,4	RF(-)
597,95	26,8	3	QQTEPGSSGSPLYSPEK	80	5,34E-10	33,0	RF(+)
597,95	26,8	3	QQTEPGSSGSPLYSPEK	80	3,70E-09	17,5	RF(-)
605,80	17,7	2	TQLAEHATPGW	80	1,44E-09	13,2	RF(+)
607,34	43,4	2	SPPLPVLESFK	90	1,70E-06	12,5	RF(+)
609,28	27,2	2	WNYVDRTTY	84	9,73E-06	8,0	RF(+)
633,76	12,3	2	PFAPCTGY	80	1,61E-09	24,7	RF(+)
633,76	12,3	2	PFAPCTGY	80	7,00E-09	15,5	RF(-)
647,33	21,6	2	TVLHQDRVYY	81	2,74E-09	6,4	RF(+)
656,65	22,8	3	NGK	82	1,30E-09	23,8	RF(+)
657,84	18,0	2	SLLHQDFGVNGK	81	5,42E-10	19,5	RF(+)
657,84	18,0	2	SLLHQDFGVNGK	81	9,53E-09	11,8	RF(-)
671,84	17,7	2	TVLHQDYAPNGK	85	4,86E-10	11,5	RF(+)
671,84	17,7	2	TVLHQDYAPNGK	85	1,30E-09	9,1	RF(-)
671,86	16,5	2	NLALHHPNVAEK	95	2,61E-08	5,4	RF(-)
674,33	18,6	3	ATLVFCDQVTHEGSTVEK	80	1,06E-08	5,8	RF(+)
675,58	26,2	4	YPSRTPEVTCVVVDVSHGEDPEVK	81	1,50E-09	121,6	RF(+)
675,58	26,2	4	YPSRTPEVTCVVVDVSHGEDPEVK	81	1,10E-09	51,7	RF(-)
675,58	25,0	4	YPSRTPEVTCVVVDVSHGEDPEVK	81	3,48E-10	12,6	RF(+)
675,58	25,0	4	YPSRTPEVTCVVVDVSHGEDPEVK	81	1,99E-09	8,3	RF(-)
682,82	31,7	2	FGGWYVDRTTY	88	3,48E-10	7,8	RF(+)
686,82	20,9	2	YTMGSPLYSPEK	82	8,28E-06	7,5	RF(+)
689,32	27,6	2	TQTGNSNLGDLAW	80	3,89E-10	5,3	RF(-)
698,81	29,4	2	FNAMYVDRTTY	80	3,89E-10	18,7	RF(+)
698,81	29,4	2	FNAMYVDRTTY	80	5,42E-10	13,5	RF(-)
711,33	31,3	2	FGNWYVNRTTY	84	4,18E-06	7,6	RF(+)
711,33	32,7	2	FGNWYVNRTTY	84	6,76E-10	16,0	RF(+)
711,33	32,7	2	FGNWYVNRTTY	84	3,04E-09	10,4	RF(-)
797,87	27,0	2	MVSHEDPEVK	89	1,94E-08	10,4	RF(+)
797,87	27,0	2	MVSHEDPEVK	89	1,58E-08	7,0	RF(-)
817,90	19,4	2	VTEPGSSGSPLYSPEK	82	5,19E-08	5,5	RF(+)
817,90	19,4	2	VTEPGSSGSPLYSPEK	82	3,04E-09	7,4	RF(-)

876,90	30,1	2	FGGTCYVDGVEVHNAK	85	4,11E-07	10,6	RF(+)
876,90	30,1	2	FGGTCYVDGVEVHNAK	85	5,90E-07	8,1	RF(-)
896,98	33,2	2	PGPSLVESGGGVVQPREK	87	3,64E-06	9,1	RF(+)

Supplemental Table 4: overview of upregulated *de novo* peptides.

RF from 27 RF(+)/anti-CCP(+) RA patients, 5 RF(-)/anti-CCP(+) RA patients, 22 RF(-)/anti-CCP(-) RA patients, 28 RF(-)/anti-CCP(-) disease controls and 4 RF(+)/anti-CCP(-) disease controls was isolated, digested into peptides and analyzed by LC-MS/MS. Data shown are upregulated *de novo* sequenced peptides. A total of 61 *de novo* sequenced peptides were upregulated when comparing RF(+) RA patients with RF(-) disease control patients samples and 36 when comparing RF(-) RA patients with RF(-) disease control patients. Underlined peptides were peptides upregulated in both the RF(+) RA patient samples as well as RF(-) RA patient samples. Peptides were considered upregulated when they had an adjusted p-value <0.05 and a FC >5.

m/z	RT	Charge	Sequence	Adj P value	FC	Group
358,23	25,8	2	LQSRVL	7,34E-09	155,5	RF(+)
358,23	25,8	2	LQSRVL	1,62E-06	61,1	RF(-)
363,67	14,4	4	TNYSPSFQGHVTL	1,61E-09	8,9	RF(+)
363,67	14,4	4	TNYSPSFQGHVTL	2,61E-08	7,3	RF(-)
375,20	14,7	2	SGVPSRF	1,92E-08	15,5	RF(+)
376,19	18,8	2	TFINAW	1,95E-07	5,3	RF(+)
376,19	18,8	2	TFINAW	3,16E-08	8,3	RF(-)
377,69	11,6	2	VGAPYTF	6,32E-09	11,6	RF(+)
380,19	12,0	3	NNFYPREAK	5,70E-09	7,8	RF(+)
380,69	15,6	2	VNPSETL	1,05E-05	6,1	RF(-)
388,93	15,2	4	ISCKGSGYRFTNY	1,79E-09	7,3	RF(+)
388,93	15,2	4	ISCKGSGYRFTNY	1,94E-08	5,6	RF(-)
391,21	14,7	3	EVVLTQSPANL	3,12E-10	22,6	RF(+)
391,21	14,7	3	EVVLTQSPANL	1,04E-09	28,7	RF(-)
394,35	12,8	6	VSSINRDGNSMDYADSVLDRF	1,61E-09	17,0	RF(+)
394,35	12,8	6	VSSINRDGNSMDYADSVLDRF	6,30E-08	10,3	RF(-)
397,94	11,7	4	YMHWVWQAPGQGL	6,74E-10	22,7	RF(+)
397,94	11,7	4	YMHWVWQAPGQGL	3,17E-08	13,0	RF(-)
398,22	13,1	3	SRVTISVDTSK	5,70E-09	19,3	RF(+)
398,88	15,6	3	YQHPPGSAPKL	3,50E-08	43,3	RF(+)
399,19	12,1	4	YCAREVEYQVLY	6,32E-09	9,7	RF(+)
401,38	13,1	5	VSSITSSSDYMYYADSVK	6,06E-10	8,5	RF(+)
401,38	13,1	5	VSSITSSSDYMYYADSVK	3,38E-09	6,3	RF(-)
402,89	15,7	3	SRVTISIDTSK	6,12E-08	16,0	RF(+)
403,22	13,7	4	YQQISGKAPNLLIY	1,61E-09	52,6	RF(+)
406,73	11,0	4	IGWVRQVPGKGLQW	8,58E-10	97,6	RF(+)
407,19	11,0	5	EWMGYVSPKNGGTDYAQK	6,01E-10	33,4	RF(+)
407,19	11,0	5	EWMGYVSPKNGGTDYAQK	8,55E-09	16,1	RF(-)
407,72	13,8	4	WTWIRQRPWKGL	7,54E-10	21,6	RF(+)
409,59	11,2	5	EWMGLVNPSGNPTNVAHGF	4,17E-09	10,0	RF(+)
416,74	19,2	2	ATGIPARF	3,23E-06	335,1	RF(+)
417,89	15,1	3	LNNFYPREAK	3,04E-09	8,1	RF(+)
419,53	11,5	3	IGTFFYSASTY	2,87E-09	14,8	RF(+)
419,53	11,5	3	IGTFFYSASTY	1,71E-07	6,5	RF(-)
420,23	22,2	2	PGQSPQLL	1,97E-06	36,8	RF(+)
421,91	15,6	3	QQRPGQAPRLL	1,11E-07	12,0	RF(+)
422,52	11,0	3	IGDITHSGSTNY	3,75E-09	74,6	RF(+)
426,26	17,8	2	PGQAPRLL	2,88E-08	11,7	RF(+)
428,27	16,9	2	LTVLGQPK	2,46E-09	14,2	RF(+)
435,57	16,9	3	GASTRATGIPARF	1,06E-08	32,3	RF(+)
437,26	18,8	2	GGGTRLTVL	4,22E-08	8,3	RF(+)
438,71	21,2	2	GDGIPDRF	1,85E-06	10,5	RF(+)
439,27	18,6	2	PGQPPRLL	9,17E-08	17,3	RF(+)
441,22	15,0	3	ASTRESGVPDRF	3,51E-08	8,7	RF(+)
441,26	17,9	2	PGQTPRLL	2,76E-06	54,5	RF(+)
443,97	13,1	4	MGWINPNSGDSKIPQK	7,04E-06	31,2	RF(+)
445,56	17,6	3	GASSRATGIPDRF	6,30E-08	6,7	RF(+)
447,22	23,8	2	SGTASVVCL	4,78E-10	64,2	RF(+)
451,22	14,7	4	LQMNNLRAEDTAVYY	5,42E-10	13,5	RF(+)
451,22	14,7	4	LQMNNLRAEDTAVYY	2,46E-09	10,6	RF(-)
452,25	14,7	2	GLQWVSTL	2,37E-07	11,2	RF(+)

455,43	19,0	5	ADSVQGRFTISRDSSRNTLY	3,04E-09	6,7	RF(+)
455,43	19,0	5	ADSVQGRFTISRDSSRNTLY	4,35E-10	7,5	RF(-)
457,23	11,3	3	ISAINGDTNHAQK	3,75E-07	20,4	RF(+)
458,76	17,2	2	VQSGAAVRK	1,42E-06	11,7	RF(+)
459,24	16,8	3	LGSNRASGVPDRF	1,47E-07	14,8	RF(+)
459,88	19,0	3	YTSNNKSYLTW	3,48E-10	5,7	RF(+)
459,88	19,0	3	YTSNNKSYLTW	3,12E-10	7,3	RF(-)
462,23	19,9	6	AMHWVRQAPGEGLEWVAVISNDGTK	1,76E-06	7,5	RF(+)
462,24	14,2	2	EWIVTIY	9,53E-09	20,4	RF(+)
470,27	12,0	2	QQLPGTAPK	3,96E-06	7,3	RF(+)
471,23	28,6	2	ISCRVSGY	2,62E-10	199,8	RF(+)
471,23	28,6	2	ISCRVSGY	1,57E-07	67,2	RF(-)
473,02	12,9	5	VSSINRDGNSMDYADSVLDRF	7,00E-09	7,8	RF(+)
473,02	12,9	5	VSSINRDGNSMDYADSVLDRF	6,45E-07	5,1	RF(-)
473,72	19,2	2	ISSSGTTMY	9,51E-09	18,5	RF(+)
473,72	19,2	2	ISSSGTTMY	1,23E-07	10,4	RF(-)
473,75	15,2	2	IMTRDTPL	3,51E-08	10,7	RF(+)
474,27	20,8	2	RTVAAPSVF	5,13E-09	7,4	RF(+)
476,27	17,6	3	YQQRPGQAPRLL	1,62E-06	8,1	RF(+)
477,24	19,5	4	ISTSSSTISYADSVKGRF	9,53E-09	9,7	RF(+)
478,91	15,8	3	DVSNRASGIPDRF	2,39E-06	13,5	RF(+)
479,75	16,2	2	IYYADSVK	1,69E-06	23,9	RF(+)
485,48	11,1	4	CQSYDNSLIYVFGSGTK	1,79E-09	12,4	RF(+)
485,48	11,1	4	CQSYDNSLIYVFGSGTK	5,19E-08	6,9	RF(-)
494,76	19,1	2	SLTPEQWK	6,32E-09	7,9	RF(+)
496,77	18,8	2	LLIYDNNK	8,28E-06	8,9	RF(+)
501,65	11,5	5	VVYDDRDRPSGIPERISGSNSGK	2,74E-09	43,2	RF(+)
501,65	11,5	5	VVYDDRDRPSGIPERISGSNSGK	1,49E-07	6,6	RF(-)
502,77	17,9	2	SASVGDRVTL	9,53E-09	17,2	RF(+)
503,76	31,5	2	SGTASVVCLL	1,48E-09	39,5	RF(+)
508,74	11,1	4	EWMGYVSPKNGGTDYAQK	1,01E-06	8,3	RF(+)
511,73	11,2	4	EWMGLVNPSGNPTNVAHGF	5,19E-08	5,8	RF(+)
511,77	22,2	2	FDISPPVTF	9,25E-08	9,8	RF(+)
515,77	14,8	2	RPSGVPDRF	3,64E-06	5,7	RF(+)
515,78	17,0	2	SLSPGERATL	3,18E-08	9,0	RF(+)
518,24	15,2	3	ISCKGSGYRFTNY	3,89E-10	9,4	RF(+)
518,24	15,2	3	ISCKGSGYRFTNY	1,04E-09	5,9	RF(-)
518,26	15,5	3	LQGRVTMTADESTK	1,02E-07	5,7	RF(+)
525,27	12,9	2	VPADTLRNY	2,74E-09	5,8	RF(+)
526,27	10,6	3	NNWPRGTFGQGTKL	1,49E-07	6,7	RF(+)
535,26	28,3	2	FGSRSGTSASL	1,11E-08	13,9	RF(+)
537,93	19,5	3	VQMNSLRAEDTALY	5,42E-10	10,7	RF(+)
537,93	19,5	3	VQMNSLRAEDTALY	1,61E-09	8,8	RF(-)
538,60	14,9	3	ACEVTHQGLSSPVTK	1,16E-09	25,2	RF(+)
543,30	13,8	3	WTWIRQRPWKGL	4,17E-09	7,3	RF(+)
544,29	27,1	2	EVQLVESGGGL	1,85E-06	68,1	RF(+)
551,29	22,4	3	IYDASNRATGIPARF	3,49E-06	14,0	RF(+)
551,30	25,2	2	LSLTPEQWK	4,71E-08	6,5	RF(+)

551,80	15,6	2	YQQLPGTAPK	3,13E-07	6,5	RF(+)
553,23	11,2	3	ISCQGSGYSFTTYW	3,18E-08	22,9	RF(+)
553,27	15,3	3	LAWYQQKPGQPPQL	6,94E-08	8,1	RF(+)
554,48	19,9	5	AMHWVRQAPGEGLEWVAVISNDGTK	1,79E-07	6,5	RF(+)
556,61	16,8	3	LQMNSLRAEDRAVY	6,48E-07	11,8	RF(+)
560,63	24,8	3	IYDASNRATGIPVRF	9,72E-08	149,8	RF(+)
560,63	24,8	3	IYDASNRATGIPVRF	2,99E-07	11,8	RF(-)
565,78	15,1	2	QSPRWGQGTL	1,61E-09	10,0	RF(+)
565,78	15,1	2	QSPRWGQGTL	3,04E-09	7,2	RF(-)
569,78	12,0	2	NNFYPREAK	3,38E-09	10,9	RF(+)
569,79	20,6	2	SLRAEDTALY	5,39E-07	8,0	RF(+)
571,26	13,0	3	SYSCQVTHEGSTVEK	6,30E-08	9,3	RF(+)
572,62	18,0	3	VKTSETLSLTCAVSGY	1,16E-09	28,3	RF(+)
572,62	18,8	3	RAGDTAVYYCARGKK	8,20E-07	393,3	RF(+)
576,80	21,4	2	SLRVEDTAVY	4,71E-08	9,8	RF(+)
576,80	30,8	2	KNGETIYAEK	5,03E-11	317,8	RF(+)
576,80	30,8	2	KNGETIYAEK	4,19E-10	135,8	RF(-)
577,27	20,3	2	DIWGQGTMVF	3,89E-10	34,2	RF(+)
577,27	20,3	2	DIWGQGTMVF	8,41E-10	22,9	RF(-)
577,27	21,7	2	DIWGQGTMVF	6,76E-10	9,0	RF(+)
577,27	21,7	2	DIWGQGTMVF	2,88E-08	6,4	RF(-)
578,95	26,9	3	QGRVTFTADTSTSTVY	5,42E-10	10,6	RF(+)
578,95	26,9	3	QGRVTFTADTSTSTVY	5,70E-09	7,1	RF(-)
579,32	29,3	2	EIVLTQSPGTL	6,81E-08	16,8	RF(+)
580,29	29,1	2	ENVLTQSPGTL	3,12E-10	10,2	RF(+)
580,29	29,1	2	ENVLTQSPGTL	3,12E-10	12,3	RF(-)
586,33	30,1	2	QIVLTQSPATL	1,06E-06	259,5	RF(+)
587,31	20,6	2	IFPPSDEQLK	4,17E-09	10,2	RF(+)
593,55	24,6	4	IFAASTLQSGVPSKFSGSGSGTHF	7,63E-06	7,5	RF(+)
596,83	13,1	2	SRVTISVDTSK	1,12E-08	45,2	RF(+)
601,29	14,6	3	NCLAWFQQKPGQPPK	1,06E-08	14,8	RF(+)
601,29	14,6	3	NCLAWFQQKPGQPPK	2,83E-06	9,3	RF(-)
603,11	27,2	6	INPNSGATTYAQNFQGRVTMTRDTSITTASMEL	9,89E-07	5,9	RF(+)
604,82	19,3	2	HHRLNVWVF	1,82E-10	136,6	RF(+)
604,82	19,3	2	HHRLNVWVF	1,82E-10	166,7	RF(-)
605,78	20,8	2	ITYSGNTYYK	2,21E-09	26,0	RF(+)
605,78	20,8	2	ITYSGNTYYK	3,38E-09	17,5	RF(-)
606,29	30,9	2	LTGYYEGYVF	3,04E-09	5,5	RF(+)
606,29	30,9	2	LTGYYEGYVF	1,16E-09	6,5	RF(-)
606,30	23,3	4	RAEDTAVYYCAKVPAATIPYY	6,08E-06	13,4	RF(+)
611,78	21,7	2	DIQMTQSPSSL	5,72E-08	12,4	RF(+)
619,81	28,4	2	RLEPEDFAVY	3,13E-07	8,0	RF(+)
624,57	24,8	4	SASVGDSVTISCRASQDIGKSLAW	2,21E-09	9,9	RF(+)
624,57	24,8	4	SASVGDSVTISCRASQDIGKSLAW	8,60E-09	6,7	RF(-)
626,33	15,0	2	LNNFYPREAK	2,46E-09	16,7	RF(+)
626,63	18,8	3	GSGSLMGHWGQGTLVTVSS	9,37E-10	13,1	RF(+)
626,82	11,5	4	VVYDDRDRPSGIPERISGSNSGK	2,74E-09	44,7	RF(+)
626,82	11,5	4	VVYDDRDRPSGIPERISGSNSGK	6,45E-07	7,5	RF(-)

632,30	16,5	2	YSGSIYYNPSL	3,07E-06	8,4	RF(+)
634,31	17,6	2	YCMQTIQLPL	3,38E-09	15,4	RF(+)
634,33	13,3	2	TQPPSVSAAPGQK	2,16E-07	14,7	RF(+)
635,98	19,5	3	YCARVRVDIVSQNYY	2,01E-06	10,5	RF(+)
638,80	19,7	2	VYACEVTHQGL	4,17E-09	33,7	RF(+)
643,85	18,3	2	QVQLVQSGAEVK	2,60E-07	8,7	RF(+)
644,31	22,1	2	TSVTPADTAVYY	1,02E-07	17,1	RF(+)
650,33	15,6	2	HISRDDSKQTL	5,39E-07	10,5	RF(+)
652,85	16,9	2	GASTRATGIPARF	6,75E-10	283,2	RF(+)
652,85	16,9	2	GASTRATGIPARF	6,75E-10	11,9	RF(-)
653,29	39,3	2	GYYPTQIDYW	1,10E-08	467,6	RF(+)
653,29	39,3	2	GYYPTQIDYW	1,37E-08	209,1	RF(-)
657,87	23,1	2	QVQLVESGGGLVK	6,15E-09	14,3	RF(+)
661,33	15,0	2	ASTRESGVPDRF	2,33E-06	75,7	RF(+)
662,68	28,7	3	AAPSVTLFPPSSEELQANK	5,13E-09	10,0	RF(+)
662,68	30,3	3	AAPSVTLFPPSSEELQANK	4,27E-08	18,2	RF(+)
664,36	24,9	2	QVQVQQWGAGLL	2,46E-09	6,4	RF(+)
664,36	24,9	2	QVQVQQWGAGLL	2,46E-09	6,9	RF(-)
674,72	27,8	3	EVQLVQTGGGLIQPGGSLRL	1,16E-09	26,9	RF(+)
675,66	23,5	3	YGSGSLMGHWGQGTLVTVSS	2,01E-06	6,6	RF(+)
681,68	28,7	3	ANPTVTLFPPSSEELQANK	1,58E-08	13,9	RF(+)
684,81	30,2	2	YCARASARNNW	4,34E-10	30,7	RF(+)
684,81	30,2	2	YCARASARNNW	6,99E-09	19,7	RF(-)
695,86	16,1	2	TQSPSASASLGASVK	1,37E-06	25,6	RF(+)
712,66	16,7	3	VDNALQSGNSQESVTEQDSK	5,13E-09	12,1	RF(+)
713,35	39,4	2	ISDFYPGAVTVAW	8,30E-09	13,7	RF(+)
720,34	36,9	2	VSYISSSGSNIYY	2,94E-10	35,6	RF(+)
720,34	36,9	2	VSYISSSGSNIYY	3,59E-09	24,1	RF(-)
720,83	43,1	2	FSWFDPWGQGTL	1,43E-07	464,8	RF(+)
720,83	43,1	2	FSWFDPWGQGTL	1,76E-07	218,9	RF(-)
730,34	25,0	3	AQQFQARVTITADESTSTAY	7,54E-10	20,8	RF(+)
730,34	25,0	3	AQQFQARVTITADESTSTAY	8,60E-09	11,5	RF(-)
755,36	14,4	3	KVDNALQSGNSQESVTEQDSK	1,97E-07	10,1	RF(+)
758,38	19,0	3	ADSVQGRFTISRDSSRNTLY	3,12E-10	17,7	RF(+)
758,38	19,0	3	ADSVQGRFTISRDSSRNTLY	3,12E-10	15,6	RF(-)
763,86	42,4	2	CQKYNSVPPWTF	2,88E-08	113,7	RF(+)
770,39	30,7	2	TISRLEPEDFAVY	4,84E-07	19,1	RF(+)
777,40	32,2	2	ISDFYPGAVTVAWK	8,98E-06	5,9	RF(+)
794,86	22,0	2	TISRVEAGDEADYY	6,30E-08	9,6	RF(+)
806,39	19,5	2	VQMNSLRAEDTALY	8,41E-10	36,6	RF(+)
806,39	19,5	2	VQMNSLRAEDTALY	2,21E-09	25,4	RF(-)
807,40	14,9	2	ACEVTHQGLSSPVTK	2,37E-06	90,0	RF(+)
809,38	14,5	3	YAQQFQGRVTMTRDTSISTAY	2,87E-09	36,7	RF(+)
825,41	44,7	2	IYGNGNRPSGVPDRF	1,57E-06	8,2	RF(-)
829,40	15,4	2	LAWYQQKPGQPPQL	1,71E-07	352,9	RF(+)
849,91	38,3	2	VSGISAGGTSTFDADSVK	2,88E-08	8,8	RF(+)
849,91	38,3	2	VSGISAGGTSTFDADSVK	8,38E-10	11,5	RF(-)
859,38	32,0	3	VTISCSGSSSNIGDNYVSWYQHL	1,30E-09	9,7	RF(+)

859,38	32,0	3	VTISCSGSSSNIGDNYVSWYQHL	9,37E-10	7,9	RF(-)
867,91	27,0	2	QGRVTFTADTSTSTVY	1,16E-09	12,5	RF(+)
867,91	27,0	2	QGRVTFTADTSTSTVY	5,19E-08	7,3	RF(-)
877,45	34,7	2	TLTISRLEPEDFAVY	6,49E-06	13,0	RF(+)
881,91	20,5	2	YLQMGSLRAEDMAVY	8,83E-08	5,7	RF(+)
881,91	20,5	2	YLQMGSLRAEDMAVY	1,88E-07	9,1	RF(-)
906,45	20,2	2	PSGVPSRFSGSGSGTAFTL	4,36E-06	Inf	RF(+)
906,45	20,3	2	PSGVPSRFSGSGSGTAFTL	3,68E-08	26,1	RF(+)
906,45	20,3	2	PSGVPSRFSGSGSGTAFTL	4,81E-06	16,2	RF(-)
910,93	18,0	2	SLTCTVSGGSIRSYFW	1,03E-09	56,2	RF(+)
918,13	30,6	3	RGLRVGSIEGDGFDIWGQGTMVIVSS	9,26E-08	16,2	RF(+)
939,44	18,9	2	GSGSLMGHWGQGTLVTVSS	1,71E-07	139,9	RF(+)
993,51	28,7	2	AAPSVTLFPPSSEELQANK	4,63E-09	11,9	RF(+)
993,51	30,1	2	AAPSVTLFPPSSEELQANK	2,19E-06	17,7	RF(+)
1022,02	28,6	2	ANPTVTLFPPSSEELQANK	1,31E-06	14,3	RF(+)
1068,49	16,7	2	VDNALQSGNSQESVTEQDSK	4,17E-09	17,3	RF(+)
1071,17	30,4	3	LMKVNSDGSHRKGDGIPDRFSGSSSGAERY	2,97E-09	6,7	RF(+)
1071,17	30,4	3	LMKVNSDGSHRKGDGIPDRFSGSSSGAERY	1,02E-09	7,6	RF(-)
1288,56	32,0	2	VTISCSGSSSNIGDNYVSWYQHL	3,11E-10	36,0	RF(+)
1288,56	32,0	2	VTISCSGSSSNIGDNYVSWYQHL	2,21E-09	24,7	RF(-)

Supplemental Table 5: overview of upregulated variable region peptides.

RF from 27 RF(+)/anti-CCP(+) RA patients, 5 RF(-)/anti-CCP(+) RA patients, 22 RF(-)/anti-CCP(-) RA patients, 28 RF(-)/anti-CCP(-) disease controls and 4 RF(+)/anti-CCP(-) disease controls was isolated, digested into peptides and analyzed by LC-MS/MS. Data shown are upregulated variable region peptides. A total of 164 sequenced peptides were upregulated when comparing RF(+) RA patients with RF(-) disease control patient samples and 51 when comparing RF(-) RA patients with RF(-) disease control patient samples. Underlined peptides were peptides upregulated in both the RF(+) RA patient samples as well as RF(-) RA patient samples. Peptides were considered upregulated when they had an adjusted p-value <0.05 and a FC >5.

Sequence	m/z	RT	Mass	Charge	Adj_p_val	FC
QVQLVESGGGLVK	657,87	23,1	1313,72	2	6,15E-09	14,3
TSVTPADTAVYY	644,31	22,1	1286,61	2	1,02E-07	17,1
QVQLVQSGAEVK	643,85	18,3	1285,69	2	2,60E-07	8,7
NNFYPREAK	380,19	12,0	1137,56	3	5,70E-09	7,8
LNNFYPREAK	417,89	15,1	1250,64	3	3,04E-09	8,1
PGQAPRLL	426,26	17,8	850,50	2	2,88E-08	11,7
GASSRATGIPDRF	445,56	17,6	1333,67	3	6,30E-08	6,7
RTVAAPSVF	474,27	20,8	946,52	2	5,13E-09	7,4
YQQRPGQAPRLL	476,27	17,6	1425,78	3	1,62E-06	8,1
SGTASVVCLL	503,76	31,5	1005,52	2	1,48E-09	39,5
SLSPGERATL	515,78	17,0	1029,54	2	3,18E-08	9,0
ACEVTHQGLSSPVTK	538,60	14,9	1612,79	3	1,16E-09	25,2
IFPPSDEQLK	587,31	20,6	1172,61	2	4,17E-09	10,2
LNNFYPREAK	626,33	15,0	1250,64	2	2,46E-09	16,7
TISRLEPEDFAVY	770,39	30,7	1538,76	2	4,84E-07	19,1
VDNALQSGNSQESVTEQDSK	1068,49	16,7	2134,96	2	4,17E-09	17,3
EIVLTQSPGTL	579,32	29,3	1156,63	2	6,81E-08	16,8
QIVLTQSPATL	586,33	30,1	1170,65	2	1,06E-06	259,5
TISRVEAGDEADYY	794,86	22,0	1587,70	2	6,30E-08	9,6
GASTRATGIPARF	435,57	16,9	1303,70	3	1,06E-08	32,3
LTVLGQPK	428,27	16,9	854,52	2	2,46E-09	14,2
LLIYDNNK	496,77	18,8	991,53	2	8,28E-06	8,9

Supplemental Table 6: Ig variable region-derived peptides significantly upregulated in RF(+)/anti-CCP(+) RA patients compared to all controls in two independent experiments.

Peptides identified as variable region-derived peptides were selected and the normalized abundance was used to perform Kruskal Wallis testing followed by a post-hoc Dunn's test. The mean abundance in RA patients and in control patients was used to calculate fold changes. Peptides with identical sequence and upregulation, defined as p-value <0.05 and FC >5 in the proof-of-concept experiment and an adjusted p-value <0.05 and FC >5 in the main experiment, with p-value and fold change shown in the table extracted from the main experiment. m/z = mass to charge.

A)

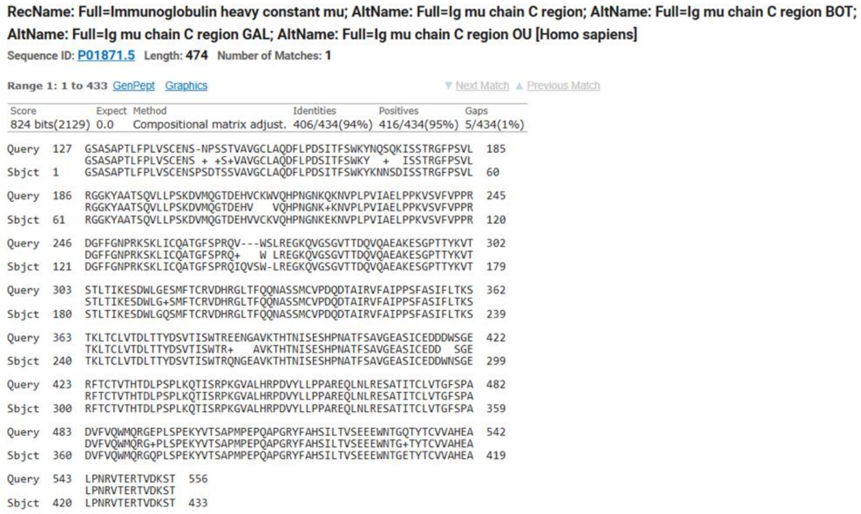

RecName: Full=Immunoglobulin heavy constant mu; AltName: Full=Ig mu chain C region; AltName: Full=Ig mu chain C region BOT; AltName: Full=Ig mu chain C region GAL; AltName: Full=Ig mu chain C region OU [Homo sapiens]

Sequence ID: P01871.5 Length: 474 Number of Matches: 1

Range 1: 1 to 433 GenPept Graphics ▼ Next Match ▲ Previous Match

Score	Expect	Method	Identities	Positives	Gaps
824 bits(2129)	0.0	Compositional matrix adjust.	406/434(94%)	416/434(95%)	5/434(1%)

```
Query  127  GSASAPTLFPLVSCENS-NPSSTVAVGCLAQDFLPDSITFSWKYNQSQKISSTRGFPSVL  185
            GSASAPTLFPLVSCENS + +S+VAVGCLAQDFLPDSITFSWKY  +  ISSTRGFPSVL
Sbjct  1    GSASAPTLFPLVSCENSPSDTSSVAVGCLAQDFLPDSITFSWKYKNNSDISSTRGFPSVL  60

Query  186  RGGKYAATSQVLLPSKDVMQGTDEHVCKWVQHPNGNKQKNVPLPVIAELPPKVSVFVPPR  245
            RGGKYAATSQVLLPSKDVMQGTDEHV   VQHPNGNK+KNVPLPVIAELPPKVSVFVPPR
Sbjct  61   RGGKYAATSQVLLPSKDVMQGTDEHVVCKVQHPNGNKEKNVPLPVIAELPPKVSVFVPPR  120

Query  246  DGFFGNPRKSKLICQATGFSPRQV---WSLREGKQVGSGVTTDQVQAEAKESGPTTYKVT  302
            DGFFGNPRKSKLICQATGFSPRQ+   W LREGKQVGSGVTTDQVQAEAKESGPTTYKVT
Sbjct  121  DGFFGNPRKSKLICQATGFSPRQIQVSW-LREGKQVGSGVTTDQVQAEAKESGPTTYKVT  179

Query  303  STLTIKESDWLGESMFTCRVDHRGLTFQQNASSMCVPDQDTAIRVFAIPPSFASIFLTKS  362
            STLTIKESDWLG+SMFTCRVDHRGLTFQQNASSMCVPDQDTAIRVFAIPPSFASIFLTKS
Sbjct  180  STLTIKESDWLGQSMFTCRVDHRGLTFQQNASSMCVPDQDTAIRVFAIPPSFASIFLTKS  239

Query  363  TKLTCLVTDLTTYDSVTISWTREENGAVKTHTNISESHPNATFSAVGEASICEDDDWSGE  422
            TKLTCLVTDLTTYDSVTISWTR+   AVKTHTNISESHPNATFSAVGEASICEDD  SGE
Sbjct  240  TKLTCLVTDLTTYDSVTISWTRQNGEAVKTHTNISESHPNATFSAVGEASICEDDWNSGE  299

Query  423  RFTCTVTHTDLPSPLKQTISRPKGVALHRPDVYLLPPAREQLNLRESATITCLVTGFSPA  482
            RFTCTVTHTDLPSPLKQTISRPKGVALHRPDVYLLPPAREQLNLRESATITCLVTGFSPA
Sbjct  300  RFTCTVTHTDLPSPLKQTISRPKGVALHRPDVYLLPPAREQLNLRESATITCLVTGFSPA  359

Query  483  DVFVQWMQRGEPLSPEKYVTSAPMPEPQAPGRYFAHSILTVSEEEWNTGQTYTCVVAHEA  542
            DVFVQWMQRG+PLSPEKYVTSAPMPEPQAPGRYFAHSILTVSEEEWNTG+TYTCVVAHEA
Sbjct  360  DVFVQWMQRGQPLSPEKYVTSAPMPEPQAPGRYFAHSILTVSEEEWNTGETYTCVVAHEA  419

Query  543  LPNRVTERTVDKST  556
            LPNRVTERTVDKST
Sbjct  420  LPNRVTERTVDKST  433
```

B)

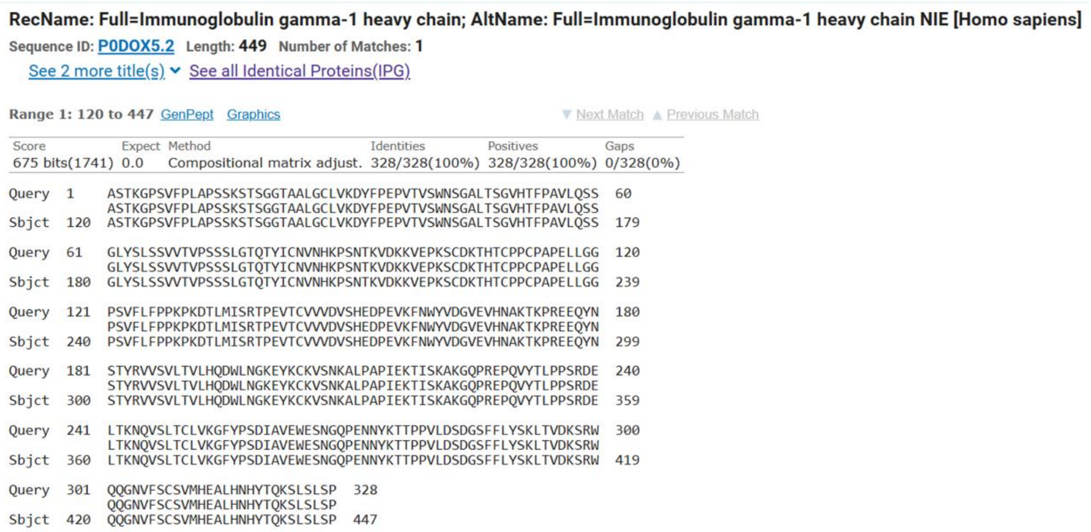

RecName: Full=Immunoglobulin gamma-1 heavy chain; AltName: Full=Immunoglobulin gamma-1 heavy chain NIE [Homo sapiens]

Sequence ID: P0DOX5.2 Length: 449 Number of Matches: 1

See 2 more title(s) See all Identical Proteins(IPG)

Range 1: 120 to 447 GenPept Graphics ▼ Next Match ▲ Previous Match

Score	Expect	Method	Identities	Positives	Gaps
675 bits(1741)	0.0	Compositional matrix adjust.	328/328(100%)	328/328(100%)	0/328(0%)

```
Query  1    ASTKGPSVFPLAPSSKSTSGGTAALGCLVKDYFPEPVTVSWNSGALTSGVHTFPAVLQSS  60
            ASTKGPSVFPLAPSSKSTSGGTAALGCLVKDYFPEPVTVSWNSGALTSGVHTFPAVLQSS
Sbjct  120  ASTKGPSVFPLAPSSKSTSGGTAALGCLVKDYFPEPVTVSWNSGALTSGVHTFPAVLQSS  179

Query  61   GLYSLSSVVTVPSSSLGTQTYICNVNHKPSNTKVDKKVEPKSCDKTHTCPPCPAPELLGG  120
            GLYSLSSVVTVPSSSLGTQTYICNVNHKPSNTKVDKKVEPKSCDKTHTCPPCPAPELLGG
Sbjct  180  GLYSLSSVVTVPSSSLGTQTYICNVNHKPSNTKVDKKVEPKSCDKTHTCPPCPAPELLGG  239

Query  121  PSVFLFPPKPKDTLMISRTPEVTCVVVDVSHEDPEVKFNWYVDGVEVHNAKTKPREEQYN  180
            PSVFLFPPKPKDTLMISRTPEVTCVVVDVSHEDPEVKFNWYVDGVEVHNAKTKPREEQYN
Sbjct  240  PSVFLFPPKPKDTLMISRTPEVTCVVVDVSHEDPEVKFNWYVDGVEVHNAKTKPREEQYN  299

Query  181  STYRVVSVLTVLHQDWLNGKEYKCKVSNKALPAPIEKTISKAKGQPREPQVYTLPPSRDE  240
            STYRVVSVLTVLHQDWLNGKEYKCKVSNKALPAPIEKTISKAKGQPREPQVYTLPPSRDE
Sbjct  300  STYRVVSVLTVLHQDWLNGKEYKCKVSNKALPAPIEKTISKAKGQPREPQVYTLPPSRDE  359

Query  241  LTKNQVSLTCLVKGFYPSDIAVEWESNGQPENNYKTTPPVLDSDGSFFLYSKLTVDKSRW  300
            LTKNQVSLTCLVKGFYPSDIAVEWESNGQPENNYKTTPPVLDSDGSFFLYSKLTVDKSRW
Sbjct  360  LTKNQVSLTCLVKGFYPSDIAVEWESNGQPENNYKTTPPVLDSDGSFFLYSKLTVDKSRW  419

Query  301  QQGNVFSCSVMHEALHNHYTQKSLSLSP  328
            QQGNVFSCSVMHEALHNHYTQKSLSLSP
Sbjct  420  QQGNVFSCSVMHEALHNHYTQKSLSLSP  447
```

Supplemental Figure 13: comparison of different Ig isotype Uniprot accesions using the Uniprot BLAST tool (extracted using NCBI Blast tool at https://blast.ncbi.nlm.nih.gov/). Illustration of the similarity of sequences of multiple Uniprot hits through database searches for identical immunoglobulin subclasses. A) Comparison of immunoglobulin heavy constant mu chain accesions (P0DOX6 as subject versus P01871 as query). B) Comparison of immunoglobulin 1 heavy constant chain accesions (P0DOX5 as query versus P01857 as subject).

	Total peptides	Unique peptides
IgA1	42	15
IgA2	43	3
IgG1	79	21
IgG2	55	6
IgG3	62	12
IgG4	59	8
IgM	85	16

Supplemental Table 7: Overview of total number of peptides used for quantification.

List of total peptides and unique peptides used for label free protein quantification.
